# Interactive Effects of Copper Contamination and Salinization Across Multiple Genotypes of *Daphnia magna*

**DOI:** 10.1002/ece3.72446

**Published:** 2025-11-05

**Authors:** Andrea Michelle Hernandez Villatoro, Jeremy J. Piggott, Adam P. Ryan, Pepijn Luijckx, Charlotte Carrier‐Belleau

**Affiliations:** ^1^ Department of Zoology, School of Natural Sciences Trinity College Dublin Dublin Ireland; ^2^ Department of Botany, School of Natural Sciences Trinity College Dublin Dublin Ireland

**Keywords:** copper, Daphnia, genotype, multiple stressors, salinization

## Abstract

Understanding how organisms respond to multiple environmental stressors is essential for predicting ecosystem impacts in the face of increasing anthropogenic pressures. However, few studies have explicitly examined how genotypes of the same species respond to combined stressors, with the specific objective of disentangling variation both within and across geographic locations. In this study, we examined the individual and combined effects of copper contamination and elevated salinity on multiple genotypes of *Daphnia magna* from US and French populations. Our findings revealed that copper exposure consistently increased mortality across all genotypes, with US genotypes displaying greater sensitivity than French counterparts. Salinity stress primarily reduced fecundity, and again, US genotypes exhibited lower resilience. Under combined copper and salinity stress, however, US genotypes showed survival benefits, suggesting potential cross‐tolerance mechanisms between these stressors. Moreover, there was substantial variation in the response to both stressors within both locations. This genotype‐specific variation underscores the necessity of considering genetic factors and genotype‐specific sensitivity/tolerance in ecosystem management and conservation strategies, particularly under multiple‐stressor scenarios. Further exploration of the genetic pathways and adaptation potential driving these responses will enhance our ability to support biodiversity and ecosystem resilience amid global environmental change.

## Graphical abstract

This study examined how *Daphnia magna* genotypes from US and French populations respond to copper and salinity stress, alone and in combination. US genotypes were generally more sensitive but showed improved survival under combined stress, indicating possible cross‐tolerance mechanisms. The strong genotype‐specific variation observed—both within and between populations—underscores the importance of incorporating genetic diversity into multiple‐stressor research and ecosystem management.

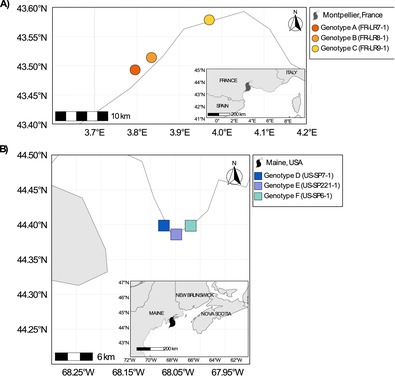

## 1 Introduction

Freshwater habitats are among the most productive ecosystems, supporting vast biodiversity and delivering essential ecosystem services (Dudgeon et al. 2006; Li and Tsigaris 2024; Strayer and Dudgeon 2010). Despite their value, freshwater ecosystems—recognized as biodiversity hotspots highly vulnerable to multiple converging anthropogenic pressures—are severely threatened by global change drivers and local human activities (Reid et al. 2019). Consequently, biodiversity declines in freshwater habitats are more pronounced than in the most impacted terrestrial ecosystems (Sala et al. 2000). Key threats include changes in water chemistry (e.g., acidification, eutrophication, and metal contamination; Greaver et al. 2016; Smith 2003), physical alterations (e.g., land‐use change, climate change, increased UV radiation; Altshuler et al. 2011; Häder et al. 2007), invasive species (Koehn 2004; Rahel 2002), and resource overextraction (Dudgeon et al. 2006; Strayer and Dudgeon 2010), all of which can degrade ecosystem function and jeopardize the services these ecosystems provide (Altshuler et al. 2011). For example, the increased presence of heavy metals such as copper (Cu), which is widely used in industry and agriculture, can accumulate to toxic levels in freshwater habitats (Gledhill et al. 1997; Zhu and Logan 2014) and disrupt physiological processes, leading to increased mutation rates and ultimately reducing population fitness, resilience, and ecosystem functioning (De Schamphelaere and Janssen 2006; Heugens et al. 2001; Osborne et al. 2020). Indeed, elevated copper concentrations have reduced primary production (e.g., macrophyte growth) and organic matter decomposition in outdoor experimental ponds (Saqira et al. 2024) and decreased bacterial diversity in freshwater microcosms (Yuan et al. 2020)—all critical, interrelated processes that drive nutrient cycling and support essential ecosystem services (Saqira et al. 2024). Additionally, rapid and extensive freshwater salinization, due to both climate change and human activities, is escalating at an unprecedented rate. Some drivers of freshwater ecosystem salinization include, for example, fluctuations in freshwater flow (Castillo et al. 2018; Jeppesen et al. 2020), agricultural practices (Jeppesen et al. 2020), sea‐level rise, land clearance (Herbert et al. 2015), and the widespread use of road salt (Castillo et al. 2018). The salinization of freshwater ecosystems has been shown to affect invertebrate species—particularly cladocerans, a key group within freshwater zooplankton—by impacting survival, reducing reproduction, decreasing body size, and delaying development (Arnott et al. 2020; Brown and Yan 2015; Li et al. 2020; Coldsnow et al. 2017). These effects can be largely attributed to the fact that exposure to high NaCl levels forces cladocerans to allocate energy toward repairing cellular damage and maintaining osmotic balance, thereby reducing energy available for other vital functions (Arnott et al. 2020; Griffith 2017). While both copper and salinity may act individually, they can also co‐occur in aquatic habitats, leading to unexpected biological responses in freshwater organisms. Although most studies examine these stressors in isolation, a few have investigated their combined effects, demonstrating impacts on species such as the zebrafish (Santos et al. 2021) and the galaxiid fish *Galaxias maculatus* (Glover et al. 2016).

The simultaneous presence of multiple stressors in freshwater ecosystems can lead to complex, often unpredictable interactions, where stressors' effects may be amplified or mitigated (Crain et al. 2008; Piggott et al. 2015) and impact all levels of biological complexity, from organisms to whole ecosystems. However, multiple interacting stressors often produce varied effects across organizational levels, with impacts on populations and ecosystems sometimes diverging from those observed in individuals (Galic et al. 2018). This variation underscores the need to understand the mechanistic underpinnings that drive these complex interactions (Schäfer et al. 2023; Schäfer and Piggott 2018). Responses to interacting stressors span various mechanisms across biological scales. At the organismal level, physiological responses (e.g., metabolic adjustments, osmoregulation) and cellular and molecular defenses (e.g., antioxidant systems) play critical roles (Heugens et al. 2001), while behavioral adaptations such as altered feeding and reproduction also contribute to stress resilience (Agatz et al. 2013). Population‐level responses include shifts in population dynamics, local extinctions, or range shifts (Vinebrooke et al. 2004; Ficke et al. 2007). At the community level, changes in species interactions and disruptions to food webs impact ecosystem structure and function (Crain et al. 2008; Jackson et al. 2016). Ultimately, genetic factors shape organisms' responses to environmental stressors (Aubin‐Horth and Renn 2009). Specifically, the trait values expressed in different environments (i.e., reaction norms) may vary among genotypes, indicating that genetic variation, together with the strength of selection, determines whether selection on a trait produces an evolutionary response (Aubin‐Horth and Renn 2009). However, the genetic mechanisms underlying responses to multiple simultaneous stressors, and which changes in gene expression correspond to plastic responses, remain largely unexplored (Aubin‐Horth and Renn 2009; Brasseur et al. 2022; Cuenca‐Cambronero et al. 2021). Moreover, ecological studies often focus on short timescales (days to weeks), during which organisms may tolerate stress through physiological acclimation (Brasseur et al. 2022). As a result, observed responses may underestimate the true negative effects of stressors. In contrast, changes in gene expression provide an immediate indication of organismal stress and can capture effects on short temporal scales. Understanding the genetic mechanisms underlying multiple‐stressor responses is therefore crucial for linking organismal plasticity to broader community‐ and ecosystem‐level impacts in aquatic environments.

A critical step in understanding these genetic mechanisms is to examine whether different genotypes of the same species respond similarly or differently to individual and combined stressors across and within locations (Barrett and Schluter 2008; Hoffmann and Sgrò 2011). Few studies have examined how aquatic organisms show differences in genetic structure across habitats (Hebert 1978). Most of this work, however, has focused on single‐stressor scenarios, with only a handful of examples addressing multiple stressors (but see Cuenca‐Cambronero et al. 2021, Fernandez‐Figueroa and Wilson 2022). Moreover, the majority of these studies focus on intrapopulation variation among genotypes (Delnat et al. 2022) and very few studies explicitly aim to disentangle responses within and across locations to these environmental changes. Investigating the responses of diverse genetic lineages across different locations to multiple stressors can reveal if consistent genetic and physiological pathways underlie resilience, offering insights into universal mechanisms organisms may use to cope with environmental challenges (Vinebrooke et al. 2004; Hoffmann and Sgrò 2011; Somero 2010). Therefore, this study aims to assess the fitness responses of multiple genotypes of *Daphnia magna* from two locations to copper contamination and elevated salinity under controlled laboratory conditions, with the goal of determining whether individual and combined stressor effects are consistent across genotypes within and across locations. To date, this is the first study to investigate the individual and combined effects of copper contamination and elevated salinity across multiple *D. magna* genotypes, both within and among locations.

The freshwater microcrustacean *Daphnia* is a well‐established model organism in studies of multiple stressors, with wide applications in evolutionary biology, ecology, toxicology, and genomics (Colbourne et al. 2011). Key attributes, such as clonal reproduction, ease of laboratory culture, scalability to population studies, and short generation times, make *Daphnia* ideal for such research (Miner et al. 2012). As a key species that connects primary production to higher trophic levels, *Daphnia* plays essential ecological roles. Genetic diversity forms the foundation for population adaptation through natural selection; therefore, understanding fitness variation across *D. magna* genotypes is a critical first step toward assessing broader implications at population and ecosystem levels. Variation in genetic diversity due to the loss of less tolerant genotypes could undermine population resilience and adaptive capacity in response to multiple environmental stressors (Medina et al. 2007; Ribeiro et al. 2012).

## 2 Materials and Methods

### 2.1 Host Preparation

**FIGURE 1:**
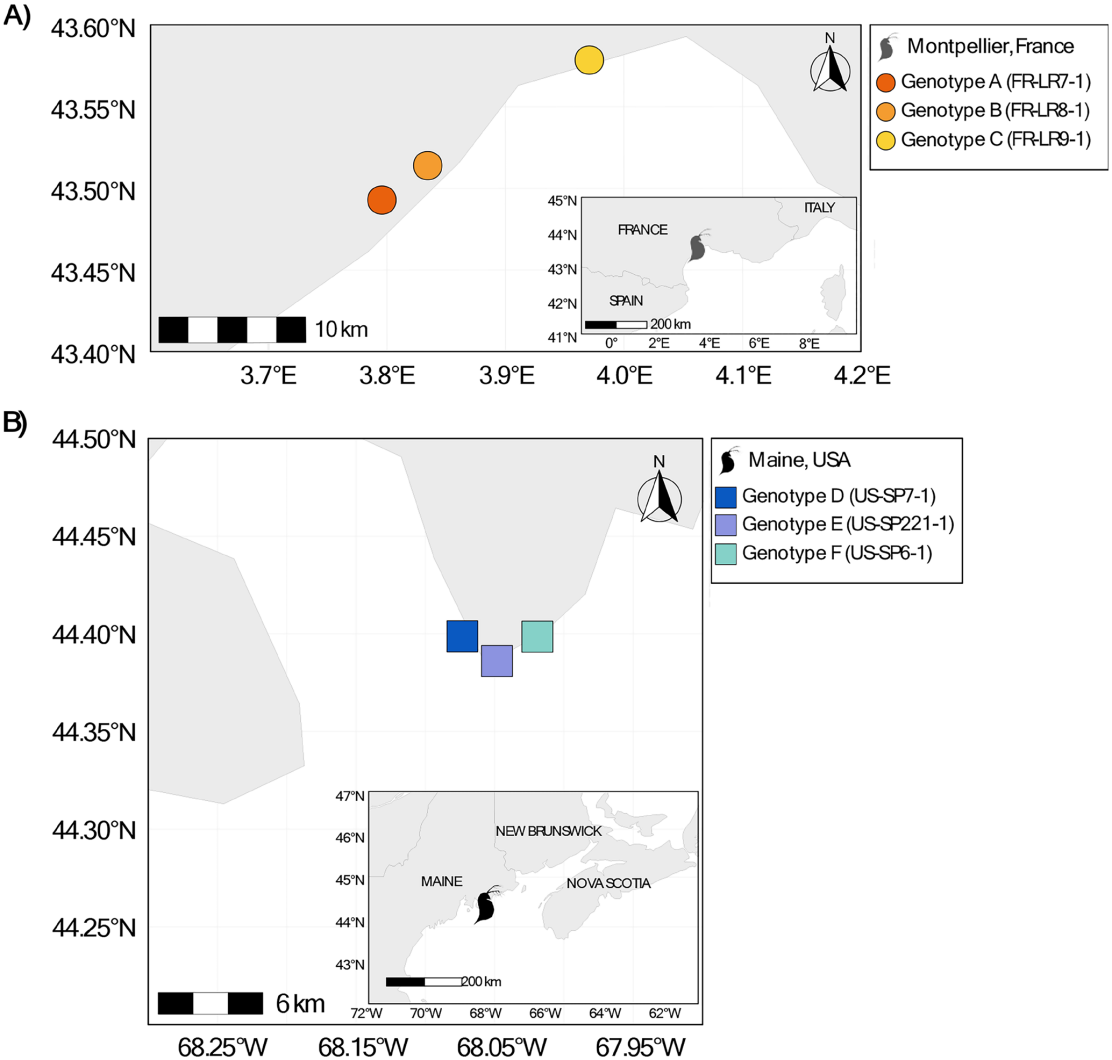
Geographic origins of selected *D. magna* genotypes from two locations: Montpellier, France, and Maine, USA (A). (B) Genotypes A, B, and C were collected from ponds near Montpellier, France. (C) Genotypes D, E, and F were collected from rock pools in Maine, USA.

The experiment involved 336 juvenile *D. magna* females from six genotypes, with three originating from France (clonal lines FR‐LR7‐1 (A), FR‐LR8‐1 (B), FR‐LR9‐1 (C)) and three from the United States (clonal lines US‐SP7‐1 (D), US‐SP221‐1 (E), US‐SP6‐1 (F); Figure 1). The six genotypes were obtained by the *Daphnia magna* Diversity Panel (Santos et al. 2024) and originated from contrasting environments (Figure 1, Table S1). Clones originating from France were collected from freshwater ponds, while clones from the USA originated from rockpools, characterized by higher fluctuating salinity. This selection allows us to compare genotypes from contrasting salinity backgrounds and assess how environmental history shapes their responses to multiple stressors. Phylogenetic analyses show a clear distinction between French and US clones, with US clones being more closely related to each other than the French ones, indicating greater genetic similarity within the US group (Figure S1). To obtain sufficient juveniles and minimize maternal effects, 30 adult *D. magna* from each genotype were collected from long‐term (50+ generations) cultures kept under standardized laboratory conditions and grown for an additional 2 weeks. Specifically, 10–12 females were placed in 400 mL glass microcosms containing 200 mL of artificial Daphnia medium with 5% of the recommended selenium dioxide concentration (ADaM; Klüttgen et al. 1994). The microcosms were maintained at 20°C and fed *ad libitum* with batch‐cultured *Acutodesmus obliquus* algae (formally known as *Scenedesmus obliquus*; Oliveira et al. 2021). Algal cultures were grown at 20°C in WC medium (Kilham et al. 1998) under nutrient‐ and light‐saturated conditions, with 40% of the culture medium replaced three times per week with fresh WC medium. Medium changes for *Daphnia* were performed twice weekly. Three days prior to the experiment, animals were transferred to new microcosms to ensure no juveniles were present. Juveniles born over the next 3 days were collected, and their sex was determined under 8×–12× magnification using a dissecting microscope to retain only females. Because *D. magna* reproduces via cyclic parthenogenesis, favorable environmental conditions promote rapid asexual reproduction, producing clonal populations of females. When environmental cues signal deteriorating conditions, male offspring are produced, marking the onset of sexual reproduction (Ebert 2022). In our experiment, male production remained low (~4%) during host preparation, indicating only limited induction of sexual reproduction. Similar low levels have been reported under favorable conditions, whereas substantially higher proportions (> 10%–20%) are typically observed when environmental stress strongly promotes male production (Ebert 2022; Gerber et al. 2018). Following sex determination, 100 females per genotype were acclimated in groups of 50 within 400 mL jars for 2 days. A total of 56 juveniles from each genotype (336 individuals) were then individually placed in 100 mL microcosms containing 50 mL of medium and randomly assigned to the experimental treatments.

### 2.2 Experimental Design and General Procedure

Individual juvenile female *D. magna* of each genotype were exposed to copper sulfate (CuSO_4_) and elevated salinity for a duration of 21 days following a full‐factorial design, resulting in four experimental treatments with 14 replicates per treatment: control conditions, copper sulfate contamination (6 μg L^−1^ CuSO_4_), elevated salinity (7 g L^−1^), and elevated salinity combined with CuSO_4_. Experimental concentrations were selected based on previous experiments investigating those two stressors on Daphnia (e.g., *D. magna* and *D. longispina* ; Arnér and Koivisto 1993; Venâncio et al. 2021; Bossuyt and Janssen 2003) and to produce minimal mortality and moderate effects on offspring production. The experimental conditions were achieved by supplementing ADaM (which naturally contains 0.33 g of salt per liter) with CuSO_4_ and synthetic sea salt (Instant Ocean, Aquarium Systems). Microcosms were organized into trays, each containing one replicate of all four treatments across the six genotypes. Trays were placed on four shelves, and organisms were maintained at 20°C. To mitigate positional effects, tray positions were randomized within and across shelves once per week. Animals were transferred to new microcosms with fresh artificial medium and corresponding treatments twice weekly to prevent the accumulation of waste products and offspring. Each individual was fed 1 mL of batch‐cultured *Scenedesmus* algae, grown in WC medium at a concentration of 8 × 10^6^ cells per mL.

### 2.3 Measurements

To evaluate the individual and combined effects of CuSO_4_ contamination and elevated salinity on different genotypes of *D. magna* , we measured survival, reproductive success, and offspring sex ratio throughout the experiment. Survival provides a direct measure of acute toxicity, while lifetime reproductive success, calculated as the total number of offspring produced, reflects sublethal effects on fecundity and overall fitness (Dang et al. 2012). Because *D. magna* reproduces asexually under favorable conditions but shifts toward sexual reproduction under environmental stress (Miner et al. 2012; Ebert 2022), we monitored the female‐to‐male ratio of offspring as an indicator of stress response and potential shifts in reproductive strategy. Mortality was recorded every 2 days, and offspring counts and sex determination were performed twice weekly during transfers to fresh mesocosms. These endpoints are widely used in ecotoxicological studies and allow for ecologically relevant assessment of both individual and population‐level effects under multiple‐stressor conditions.

### 2.4 Statistical Analyses

To assess the individual and combined effects of CuSO_4_ contamination and elevated salinity across different genotypes of *D. magna* from two locations on survival, reproductive success, and offspring sex ratio, we employed Generalized Linear Models (GLMs). Survival was modeled using a Poisson distribution, while reproductive success (offspring counts) and offspring sex ratio were analyzed using a negative binomial distribution due to the presence of overdispersion. Fixed factors in the models included CuSO_4_, salinity, and genotype nested within location, and their interactions. Post hoc comparisons were conducted using custom contrasts corrected for multiple testing using the Benjamini–Hochberg procedure. Those contrasts were directly linked to the aims of our study: that is, comparison of the responses of the different genotypes within and across locations between treatments. Due to an experimental mistake, one replicate was lost during the experiment and was excluded from the analyses. All analyses were performed in R (version 4.4.1), using the lme4 package for model fitting. We set the possibility of making a type I error to *α* = 0.05.

## 3 Results

### 3.1 Survival

**FIGURE 2:**
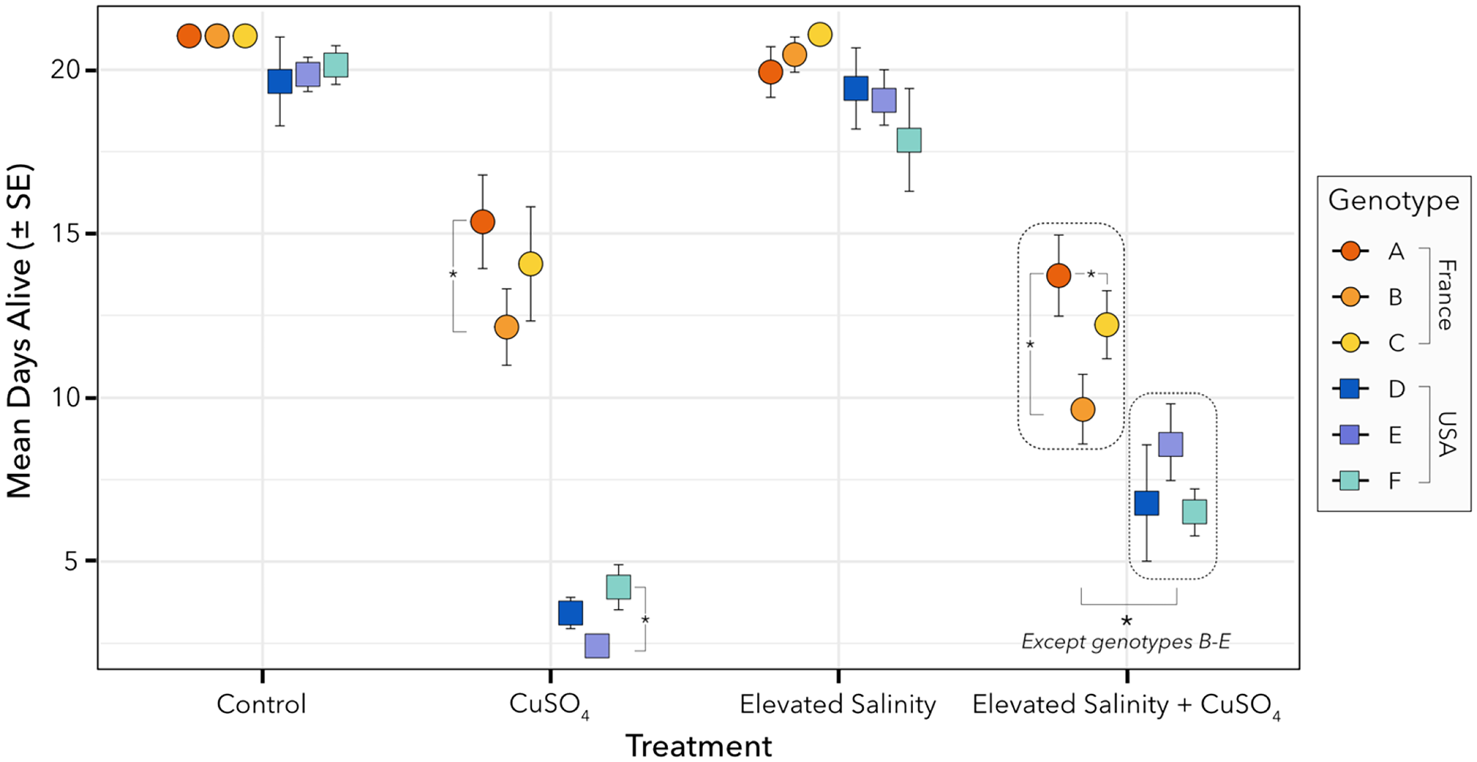
Mean days alive (±SE) of *D. magna* across experimental conditions: Control, CuSO_4_, Elevated salinity and CuSO_4_ + Elevated Salinity. Results are shown for the six genotypes (A–F) from two locations—France (genotypes A, B, C) and the USA (genotypes D, E, F). Asterisks show significant differences between genotypes within and across locations. All French genotypes differ from all USA genotypes, except for B and E, which are not significantly different.

The survival of the six *D. magna* genotypes from two locations (France and USA) exhibited distinct responses to CuSO_4_ contamination, elevated salinity, and their combination (Figure 2). Under control conditions, all genotypes responded the same across the two locations, with no differences in survival rates (*p* > 0.05 for all custom contrasts, Table S2). Elevated salinity alone had no impact on survival rates across all genotypes from both locations (GLM, df = 1, *p* > 0.9899, Table 1), with no detectable differences either within or between locations (*p* > 0.05 for all custom contrasts, Table S2). However, exposure to CuSO_4_ reduced survival compared to the control, with notable differences both within and across locations. In particular, genotypes from the USA displayed lower survival rates (mean of 3.36 days, SE = 0.52) than those from France (mean of 13.86 days, SE = 2.01; GLM, df = 1, *p* = 2.20E^−16^, Table 1). Within the French population, CuSO_4_ exposure differentially affected survival across genotypes, with a reduction in survival for genotype B compared to genotype A (custom contrast, *p* = 0.0452, Table S2). Similarly, differences were observed among the US genotypes, where genotype E had a lower survival rate compared to genotype F (Custom contrast, *p* = 0.0219, Table S2).

**TABLE 1:** Summary of Generalized Linear Model analysis: Effects of genotype, CuSO_4_, and salinity on the survival of *D. magna* .

	Df	Deviance	Residual Df	Residual deviance	*p*
NULL			332	1474.83	
Genotype	5	119.53	327	1355.30	< 2.20E‐16***
Salinity	1	0.00	326	1355.30	0.990^ns^
CuSO_4_	1	70,331	325	651.99	< 2.20E‐16***
Genotype:Salinity	5	15.07	320	636.91	0.010*
Genotype:CuSO	5	169.21	315	467.70	< 2.20E‐16***
Salinity:CuSO	1	9.23	314	458.47	0.002**
Genotype:Salinity:CuSO	5	59.05	309	399.43	1.91E‐11***

*Note:* Results are shown for degrees of freedom (Df), deviance, residual Df, residual deviance and *p*‐values. Asterisks indicate levels of statistical significance: *p < 0.05, **p < 0.01, ***p < 0.001, †0.05 ≤ *p* < 0.10; ns, not significant.

When both CuSO_4_ and elevated salinity were combined, survival responses differed between genotypes from both locations (GLM, df = 5, *p* = 1.912E^−11^, Table 1), except for genotypes B (France) and E (USA), which responded similarly. Intra‐location variability was detected within the French populations (Custom contrast; genotypes A and B, *p* = 0.0041, genotypes A and D, *p* < 0.0001, Table S2), while a marginally significant difference was found among the US genotypes (Custom contrast, genotypes E and F, *p* = 0.0785, Table S2). For the French genotypes, the combined exposure to CuSO_4_ and elevated salinity did not decrease survival compared to CuSO_4_ alone (Custom contrast, *p* > 0.05 for genotypes A, B, and C; Table S2). However, survival rates were lower compared to exposure to elevated salinity alone (Custom contrast, *p* < 0.05 for genotypes A, B, and C; Table S2). In contrast, US genotypes exhibited an increase in survival under the combined stressors compared to CuSO_4_ exposure alone (Custom contrast, *p* < 0.05 for genotypes D, E, and F; Table S2), though survival was still lower than under salinity stress alone (Custom contrast, *p* < 0.0001 for genotypes D, E, and F; Table S2). A complete summary of the GLM output, including effect sizes, as well as the means and standard errors for each treatment, is provided in Tables S3 and S4, respectively.

### 3.2 Offspring Production and Sex Ratio

**FIGURE 3:**
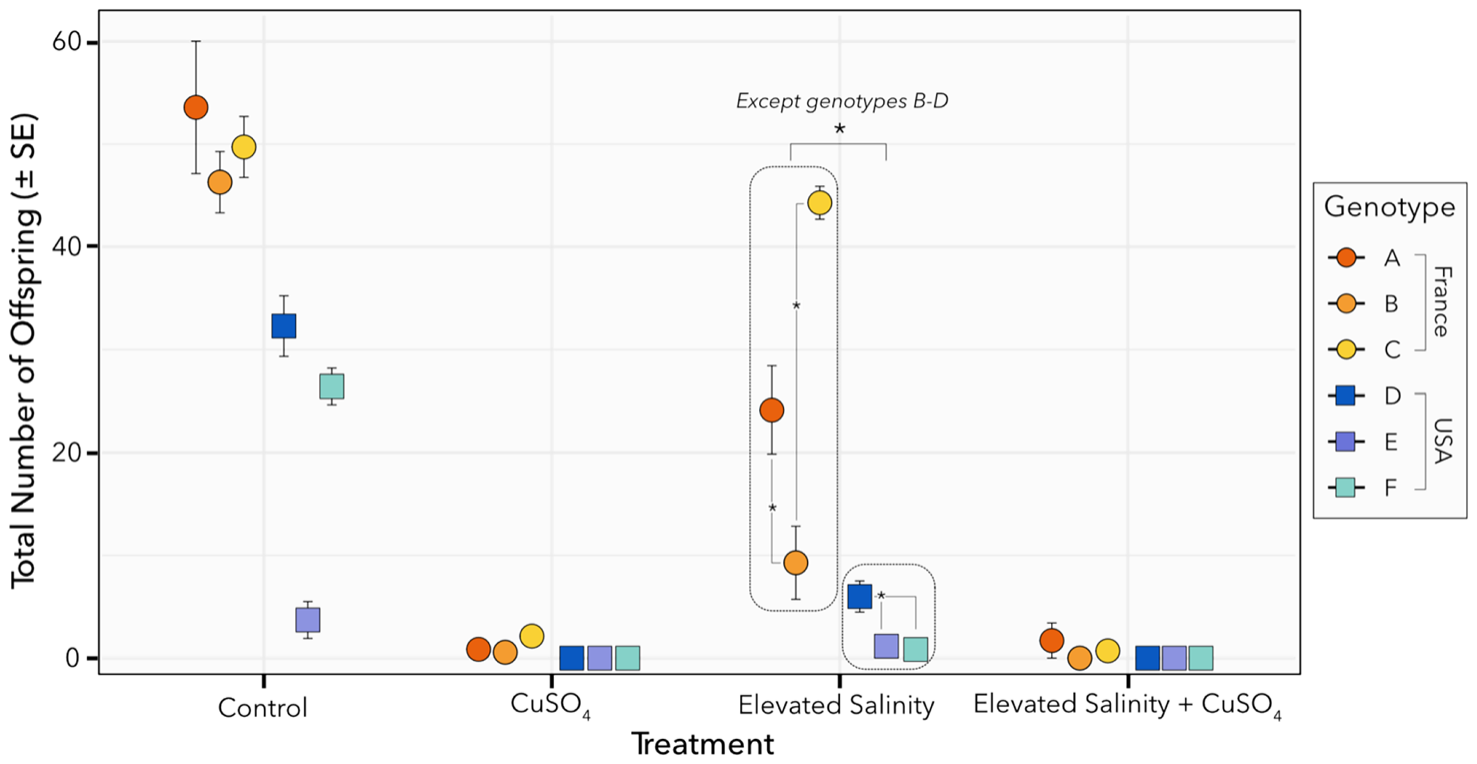
Total number of offspring (±SE) of *D. magna* across experimental conditions: Control, CuSO_4_, Elevated salinity and CuSO_4_ + Elevated Salinity. Results are shown for the six genotypes (A–F) from two locations—France (genotypes A, B, C) and USA (genotypes D, E, F). Asterisks show significant differences between genotypes within and across locations. All French genotypes differ from all USA genotypes, except for B and D, which are not significantly different.

The six *D. magna* genotypes, originating from France and the USA, displayed different fecundity responses to CuSO_4_, elevated salinity, and their combination (Figure 3). Both stressors individually reduced fecundity relative to control conditions, with CuSO_4_ having a particularly strong effect, nearly eliminating offspring production. Two‐way interactions were observed between elevated salinity and genotype (GLM, df = 5, *p* = 1.368E^−08^, Table 2) and between CuSO_4_ and genotype (GLM, df = 5, *p* = 0.00022, Table 2). Further analysis indicated intra‐location variability under elevated salinity stress in French genotypes, with differences in fecundity between genotypes A and B (Custom contrast, *p* = 0.0075, Table S5) and genotypes B and C (Custom contrast, *p* < 0.0001, Table S5). This intra‐location variability was mirrored in the offspring sex ratio, with genotypes A and B exhibiting lower female‐to‐male ratios compared to genotype C, highlighting genotype‐specific trait differences within the same location (Figure S2). However, high mortality and reduced offspring production in certain treatments (e.g., CuSO_4_ and combined CuSO_4_ with elevated salinity) limited comparative analysis across all treatments. Nevertheless, the supplementary data provide a comprehensive account of these treatment effects (Tables S6 and S7, Figure S1). Additionally, inter‐location differences also emerged under elevated salinity, as all French genotypes differed from the US genotypes, except for genotypes B and D, which did not differ (Figure 3, Table S5). Moreover, a marginally significant interaction among location, genotype, salinity, and CuSO_4_ indicates that offspring production varies between genotypes from different locations in response to the combined stressors (GLM, df = 4, *p* = 0.0543, Table 2). Finally, natural variation in offspring production was observed under control conditions, with US genotypes generally producing fewer offspring than French genotypes and exhibiting greater variability within the US location (Figure 3, Table S5). A detailed summary of the GLM output of total fecundity and female‐to‐male ratio, including effect sizes, along with the means and standard errors for each treatment, is presented in Tables S8 and S9, respectively.

**TABLE 2:** Summary of Generalized Linear Model analysis: Effects of genotype, CuSO_4_, and salinity on the total number of offspring in *D. magna* .

	Df	Deviance	Residual Df	Residual deviance	*p*
NULL			167	1375.79	
Genotype	5	264.47	162	1111.32	< 2.20E‐16***
Salinity	1	132.01	161	979.31	< 2.20E‐16***
CuSO_4_	1	715.50	160	263.81	< 2.20E‐16***
Genotype:Salinity	5	45.13	155	218.68	1.37E‐08***
Genotype:CuSO	5	24.00	150	194.68	2.20E‐04***
Salinity:CuSO	1	0.08	149	194.60	0.771^ns^
Genotype:Salinity:CuSO	5	9.29	144	185.31	0.098†

*Note:* Results are shown for degrees of freedom (Df), deviance, residual Df, residual deviance and *p*‐values.

^†^ It refers to marginally signifiant results.

## 4 Discussion

Identifying the individual and combined impacts of interacting stressors on aquatic ecosystems is crucial for understanding and predicting their effects on organisms, populations, and ecosystem dynamics. Our study revealed that both elevated salinity and CuSO_4_ significantly affected *D. magna* genotypes from both locations, both as individual stressors and in combination. Notably, we observed variability in fitness responses to these stressors within and between populations. This highlights the importance of genetic mechanisms and evolutionary history in shaping organismal responses to multiple stressors. Such variability in fitness across genotypes underscores the need for further investigation into the genetic and evolutionary processes underlying these responses. These findings have important implications for management and conservation, emphasizing the necessity of incorporating genetic diversity and stressor interactions into future ecological and conservation strategies.

### 4.1 Individual and Combined Effects of CuSO_4_ and Elevated Salinity

Across all measured responses, CuSO_4_ had a more pronounced negative impact than elevated salinity when tested as a single stressor, leading to reduced survival and offspring production in all genotypes relative to control conditions. This impact also varied by geographic origin, with genotypes from the USA experiencing greater adverse effects compared to those from France. Copper contamination is known to detrimentally affect freshwater ecosystems, including various *Daphnia* species such as *D. pulex* (Liorti et al. 2016; Lytle 1999), *D. magna* (Liu et al. 2019), and *D. longispina* (Lopes et al. 2009). The toxicity mechanisms of copper in aquatic organisms involve oxidative stress and cellular damage (Brouwer and Brouwer 1998), disruption of ion regulation (Chavez‐Crooker et al. 2003; Tellis et al. 2014), and genotoxic and epigenetic effects (Cavas et al. 2005), leading to increased mortality (Venâncio et al. 2021), impaired fecundity (Atienzar 2001), and altered behavior (Yuan et al. 2003) in *Daphnia* species. While copper contamination was the dominant stressor in our system, elevated salinity also led to reduced fitness. Although we lack detailed information on the histories of copper exposure, the observed differences in sensitivity between US and French genotypes may reflect contrasting local selective pressures. Populations exposed to metals frequently or over extended periods can evolve increased tolerance, whereas populations from historically low‐exposure habitats remain more susceptible. Rapid adaptation to copper and zinc has been documented in Daphnia, resulting in substantial between‐population variation in tolerance (Hochmuth et al. 2015).

Salinity did not impact survival rates as observed in other studies (Sun and Arnott 2022) but the fecundity of animals was reduced. Osmoregulation in *Daphnia* primarily occurs in the nuchal organs or epipodites (Aladin and Potts 1995), where ion transporters rely on Na^+^ gradients maintained by Na^+^‐K^+^‐ATPase (NKA; Péqueux 1995; Grosell 2013). Under osmotic stress, Daphnia can increase NKA activity (Bal et al. 2021), but as NKA requires ATP, this upregulation can strain the energy budget and raise metabolic rates, leading to the production of reactive oxygen species (ROS) as a by‐product of oxidative phosphorylation (Bal et al. 2021). ROS can damage cellular structures like DNA, proteins, and lipids (Barata et al. 2005), causing short‐ and long‐term negative impacts. Importantly, oxidative stress has also been directly linked to reduced reproductive performance, as ROS can impair gametes and reproductive tissues, decreasing offspring production (Barata et al. 2005), while the upregulation of antioxidant defenses and repair mechanisms diverts energy away from reproduction (Heckmann et al. 2008; Monaghan et al. 2009; Metcalfe and Alonso‐Alvarez 2010). Thus, osmotic stress can lead to suboptimal physiological conditions, disrupting energy balance and inducing cellular damage (Ghazy et al. 2009), with consequences for reproductive investment. In our experiment, although individuals tolerated elevated salinity without immediate effects on survival, these conditions adversely affected fecundity, likely through the mechanisms described above. These physiological disruptions caused by salinity stress provide important context for understanding how combined stressors, like copper contamination and elevated salinity, interact to influence biological responses and overall fitness.

When combined, copper contamination and elevated salinity altered survival outcomes compared to each stressor alone. Specifically, the combination increased survival in USA genotypes relative to copper exposure alone, while the survival of French genotypes remained unchanged. This suggests that elevated salinity may have triggered protective responses in organisms already exposed to copper for the genotypes from the USA. Exposure to one stressor can sometimes enhance tolerance to another through overlapping physiological defense mechanisms, a phenomenon known as cross‐tolerance (Pallarés et al. 2017; Todgham et al. 2005). For instance, elevated salinity can lead to ROS buildup and upregulation of antioxidant defenses, potentially enhancing tolerance to a secondary stressor (Bal et al. 2021). Conversely, the combination of both stressors decreased survival across all genotypes compared to elevated salinity alone, indicating possible cross‐susceptibility, where exposure to one stressor compromises resistance to another, leading to energy and physiological trade‐offs (Botella‐Cruz et al. 2016; Todgham et al. 2005). The toxicity of metals, particularly copper, can increase with elevated salinity, negatively impacting various freshwater and estuarine organisms, such as the copepod *Acartia tonsa* (Lauer and Bianchini 2010) and *D. magna* (Semsari and Haït‐Amar 2001). Genotype‐specific variations in response to single and combined stressors were evident in our system, highlighting the critical role of underlying genetic and evolutionary mechanisms in advancing multiple‐stressor research. This variation enhances the potential for adaptation and persistence by supplying the genetic diversity upon which natural selection can act. When environmental conditions change, some genotypes may be more tolerant and therefore maintain higher fitness, allowing populations to persist despite stress (Kawecki and Ebert 2004; Barrett and Schluter 2008). Genetic diversity also buffers populations against unpredictable and fluctuating environments, reducing the risk of extinction by ensuring that not all individuals respond identically to stressors (Forsman and Wennersten 2016; Hughes et al. 2008; Wernberg et al. 2018). This diversity in stress responses thus underpins both short‐term resilience and long‐term adaptive potential under environmental change.

### 4.2 Mechanisms Underlying Responses to Multiple Stressors and Variation Across Genotypes

There was substantial variation in fitness among genotypes, both within and across locations and habitats, suggesting that genetic and molecular mechanisms play a key role in driving differential responses to individual and combined stressors. For instance, within‐population variation was evident in the number of offspring produced by French genotypes in response to elevated salinity. In contrast, across‐population variation was observed in survival, with French and US genotypes exhibiting different responses to CuSO_4_ and the combination of CuSO_4_ and elevated salinity. Within‐population variation was observed, for example, for the number of offspring of French genotypes in response to elevated salinity, and across‐population variation was observed between French and US genotypes in response to CuSO_4_ and CuSO_4_ combined with elevated salinity for survival. Responses in aquatic organisms to multiple stressors are complex, involving molecular, cellular, and physiological pathways such as gene regulation and expression, detoxification, metabolic pathways, and DNA repair mechanisms. In our study, multiple pathways and mechanisms may underlie the responses to individual and combined stressors, as well as the variation observed both within and across locations. First, gene regulation (i.e., upregulation and downregulation of specific genes) and expression profiles enable organisms to initiate adaptive physiological responses to stress. For example, Chain et al. (2019) found that various *D. pulex* clones showed similar regulatory responses to copper contamination, with genes associated with digestion, molting, oxidative stress response, and metal binding being upregulated, while certain immune and oxygen transport genes were downregulated. However, while some gene regulation patterns are consistent in their pathway of activation, variation in expression levels can occur across genotypes, significantly influencing fitness outcomes. In their study, for instance, one *Daphnia* clone exhibited reduced upregulation of the glutathione‐S‐transferase (gst) gene, a crucial component in copper detoxification, resulting in heightened sensitivity to copper (Chain et al. 2019). Such differences in gene expression profiles among clones underscore the role of genetic variability in determining stress tolerance, where even subtle shifts in gene expression can lead to fitness consequences under environmental stress. Second, detoxification and metabolic pathways are integral to coping with pollutants and salinity fluctuations. Cytochrome P450 enzymes, for instance, play a critical role in detoxification by catalyzing the biotransformation of xenobiotics (Guengerich 2008; Zanger and Schwab 2013). Differences in the expression of P450 enzymes across genotypes and species contribute to variability in detoxification efficacy (Abdullahi et al. 2022), potentially conferring greater resilience in certain genotypes or populations under similar stress conditions. Third, genomic integrity and DNA repair mechanisms are fundamental for maintaining stability under stress. Environmental stressors such as UV radiation, pollution, and chemicals can cause DNA damage, activating pathways like nucleotide excision repair (NER), base excision repair (BER), and mismatch repair. In particular, Miner et al. (2015) demonstrated variation among *D. melanica* genotypes in their efficiency of photoenzymatic repair of UV‐induced DNA damage, potentially influencing population‐level resilience to UV stress. Other mechanisms, such as cross‐tolerance and stress‐induced cross‐resistance (Pallarés et al. 2017), transgenerational plasticity through epigenetic inheritance, maternal effects (Sun et al. 2023), and signal transduction pathways (Soetaert et al. 2007) further impact responses to environmental stressors. Factors such as rapid local adaptation (Wersebe and Weider 2023), phenotypic plasticity (Brans and De Meester 2018; Scoville and Pfrender 2010), and evolutionary processes (Cuenca‐Cambronero et al. 2021) also contribute to the observed genetic variability in stress responses across genotypes and may have contributed to tolerance (or lack thereof) to individual and combined stressors.

We observed differences between locations (France vs. USA), which may reflect distinct environmental histories and exposure to anthropogenic stressors, as well as between habitats (ponds vs. rockpools), which are characterized by differing stressor magnitudes and frequencies. These differences likely contribute to the varied stress response mechanisms observed in our study. The natural variability among genotypes in control conditions in both locations and alternate responses of different genotypes to multiple stressors suggests that genetic diversity and environmental history jointly shape reproductive capacity and stress response. Moreover, microhabitat differences within locations can also impose unique selective pressures, favoring particular genotypes (Richardson et al. 2014). Limited gene flow in local populations can also lead to micro‐evolutionary changes, where isolated subpopulations diverge over time, developing unique stress responses (Kawecki and Ebert 2004). Within a single location, phenotypic plasticity allows individuals to adjust their physiology according to immediate environmental conditions (Ghalambor et al. 2007), enabling dynamic adaptation to localized stressors. These organism‐level differences among multiple genotypes may scale up to affect higher levels of biological organization, including populations and entire ecosystems, with potential implications for the management and conservation of freshwater habitats.

### 4.3 Implications for Conservation and Management

Differences in *D. magna* responses to multiple stressors across genotypes highlight the importance of genetic diversity in shaping resilience within freshwater ecosystems, with implications for conservation and management. Indeed, in marine ecosystems, recovery following disturbances has been shown to improve in seagrass beds and kelp forests with higher genetic diversity (Reusch et al. 2005; Wernberg et al. 2018). Similarly, in both marine and terrestrial habitats, greater genetic diversity in foundation species is associated with increased biodiversity across trophic levels (Crutsinger et al. 2006; Reusch et al. 2005; Wernberg et al. 2018). Genetic diversity within *Daphnia* populations allows for a range of tolerance thresholds to environmental stressors, supporting population stability by enabling various genotypes to withstand stressors like pollution or salinity fluctuations (Loria et al. 2022; Miner et al. 2012). While our study examined only three genotypes from two locations, and therefore cannot fully capture population‐level genetic diversity, the observed genotype‐specific variation supports the idea that intraspecific differences can influence resilience. More comprehensive assessments incorporating greater numbers of genotypes or populations with contrasting levels of diversity will be necessary to evaluate how general these patterns are (Kawecki and Ebert 2004; Hughes et al. 2008). Approaches such as genome‐wide association studies (GWAS) could be particularly valuable, as they enable the identification of genetic variants associated with tolerance to stressors and provide a mechanistic link between genetic diversity and ecological performance (Orsini et al. 2013; Routtu and Ebert 2015).

Genetic diversity is particularly important in the context of ongoing climate change and increasing anthropogenic pressures, as different genotypes may be better suited to survive in specific environmental conditions or under particular stressor combinations (Miner et al. 2012). By maintaining genetically diverse populations, managers can help safeguard ecosystem resilience, as *Daphnia* species play a keystone role in freshwater food webs, linking primary production and higher trophic levels (Hebert 1978; Lampert 2006). Moreover, risk assessments for freshwater ecosystems that incorporate intraspecific diversity in stress responses are likely to be more accurate, capturing the complexity of interactions across multiple genotypes and enabling the prediction of population responses under varying environmental stressors (Baird and Barata 1998). Conservation strategies can also benefit from identifying and prioritizing habitats that support stress‐resistant genotypes or exhibit high genetic diversity, as these populations may be more capable of withstanding future environmental changes (Kawecki and Ebert 2004; Miner et al. 2012). Ultimately, an adaptive management approach that considers genetic variability within key species like *Daphnia* could be instrumental in maintaining ecosystem function and resilience, particularly as freshwater systems face the dual pressures of climate change and pollution (Miner et al. 2012).

## 5 Conclusion and Recommendation for Future Work

In conclusion, the diverse responses of *D. magna* genotypes to individual and combined environmental stressors, such as copper contamination and elevated salinity, highlight the complexity of mechanisms underlying responses to multiple stressors and emphasize the essential role of genetic diversity in species' resilience to environmental change. Patterns of sensitivity between and within populations underscore the importance of studying multiple genotypes from diverse locations and habitats to fully understand the intricacies of multiple‐stressor interactions. Focusing solely on single genotypes in experimental studies oversimplifies natural diversity, limiting our ability to generalize findings to the genetic variability found in natural populations. Our findings, which reveal differences in fitness among genotypes across and within locations, emphasize the value of investigating trait variability across multiple genotypes and mechanistic underpinnings in stressor interaction studies.

Ultimately, an organism's response to environmental stressors is influenced by its genetic makeup. While genetic factors govern both organismal responses and phenotypic plasticity to environmental drivers (Aubin‐Horth and Renn 2009), the genetic basis of responses to concurrent, multiple stressors remains largely uncharted (but see Li et al. 2023; Cline et al. 2020; Kelly et al. 2016). Interclonal variation in toxicology and multiple‐stressor ecology, particularly within model organisms such as *Daphnia*, has not been extensively studied, despite its implications for aquatic ecosystem risk assessment (Chain et al. 2019). Future research in this area should prioritize identifying the genes involved in complex stress responses to provide a clearer mechanistic understanding of stressor interactions. This knowledge will help determine whether responses are driven by combinations of genes linked to individual stressors, genetic interactions (epistasis), or a unique set of genes specifically responsive to multiple stressors. Such studies can be enhanced by exploring fitness variations across genotypes and conducting large‐scale genome‐wide studies.

By focusing on individual‐level mechanisms, findings can be extrapolated to higher biological levels, offering a better understanding of population‐ and ecosystem‐level responses to multiple environmental stressors. Additionally, research on keystone species like *Daphnia*, which inhabits a wide range of freshwater habitats (e.g., Arctic and temperate lakes, high‐elevation lakes, ephemeral ponds) and plays a critical role in linking primary production to higher trophic levels (Hebert 1978, Lampert 2006), will yield insights into the impacts of multiple‐stressor interactions within freshwater ecosystems.

## Author Contributions

**Andrea Michelle Hernandez Villatoro:** conceptualization (equal), data curation (lead), formal analysis (lead), investigation (lead), methodology (equal), visualization (lead), writing – original draft (lead), writing – review and editing (supporting). **Jeremy J. Piggott:** conceptualization (supporting), funding acquisition (equal), writing – review and editing (equal). **Adam P. Ryan:** formal analysis (supporting), visualization (supporting), writing – review and editing (supporting). **Pepijn Luijckx:** conceptualization (equal), formal analysis (supporting), funding acquisition (equal), methodology (supporting), supervision (equal), writing – original draft (supporting), writing – review and editing (supporting). **Charlotte Carrier‐Belleau:** conceptualization (equal), data curation (supporting), formal analysis (supporting), investigation (supporting), methodology (supporting), supervision (equal), visualization (supporting), writing – original draft (supporting), writing – review and editing (supporting).

## Conflicts of Interest

The authors declare no conflicts of interest.

## Supporting information


**Data S1:** ece372446‐sup‐0001‐Supinfo.docx.

## Data Availability

All data are provided in Data S1.
