# Supplementary material for: Interactive Effects of Copper Contamination and Salinization Across Multiple Genotypes of Daphnia magna
Source: Ecol Evol. 2025 Nov 5;15(11):e72446. doi: 10.1002/ece3.72446 (PMC12588729; doi:10.1002/ece3.72446)
Supplement: Supplementary file 1 — Data S1: ece372446‐sup‐0001‐Supinfo.docx. [file ECE3-15-e72446-s001.docx]

**Supplementary material for manuscript:**

Interactive effects of copper contamination and salinization across multiple genotypes of *Daphnia magna.*

Authors and affiliations: Andrea Michelle Hernandez Villatoro^a^, Jeremy J. Piggott^a^, Adam P. Ryan^b^, Pepijn Luijckx^a,1^, Charlotte Carrier-Belleau^a,1^

1. Zoology, School of Natural Sciences, Trinity College Dublin, Dublin, Ireland
2. Botany, School of Natural Sciences, Trinity College Dublin, Dublin, Ireland

Corresponding authors: Charlotte Carrier-Belleau ([carrierc@tcd.ie](mailto:carrierc@tcd.ie))

1. These authors contributed equally to this work.

Table S1: Origin, habitat, and bioclimatic variables of the six Daphnia magna clones from the Diversity Panel used in this study.

| Clone | Origin | Sampling year | Habitat | Annual Mean Temperature (°C) | Maximum Temperature of Warmest Month (°C) | Minimum Temperature of Coldest Month (°C) | Annual precipitation (mm) | Pond surface area (m2) |
| --- | --- | --- | --- | --- | --- | --- | --- | --- |
| FR-LR7-1 | France | 2017 | Pond | 15.00 | 27.40 | 3.10 | 612 | 11 310 |
| FR-LR8-1 | France | 2017 | Pond | 14.95 | 27.70 | 2.80 | 615 | 17 395 |
| FR-LR9-1 | France | 2017 | Pond | 14.93 | 27.80 | 2.80 | 639 | NA |
| US-SP7-1 | USA | 2016 | Coastal – rockpool | 6.73 | 21 | -9.80 | 1337 | NA |
| US-SP221-1 | USA | 2016 | Coastal – rockpool | 6.73 | 21 | -9.80 | 1337 | NA |
| US-SP6-1 | USA | 2016 | Coastal – rockpool | 6.73 | 21 | -9.80 | 1337 | NA |

Table S2: Custom Contrast Analysis comparing *Daphnia magna* survival across various combinations of elevated salinity and CuSO_4_ among multiple genotypes from two distinct location. Results are shown for comparisons amongst and between controls, elevated salinity (“Salinity”), CuSO_4_, and both stressors combined (“Both”) across all genotypes (A to F). Significant differences are highlighted in bold.

| **Contrast** (Treatment_Genotype) | | | **Estimate** | **SE** | **df** | **z ratio** | **p-value** |
| --- | --- | --- | --- | --- | --- | --- | --- |
| **Group 1** | **Group 2** |  |  |  |  |  |  |
| Control_A | Salinity_A |  | 0.0524 | 0.0836 | Inf | 0.627 | 0.6796 |
| Control_A | CuSO_4__A |  | 0.3129 | 0.0897 | Inf | 3.487 | **0.0012** |
| Control _A | Both_A |  | 0.4261 | 0.0928 | Inf | 4.592 | **<.0001** |
| CuSO_4__A | Salinity_A |  | -0.2606 | 0.0907 | Inf | -2.871 | **0.0091** |
| CuSO_4__A | Both_A |  | 0.1131 | 0.0993 | Inf | 1.139 | 0.4141 |
| Salinity_A | Both_A |  | 0.3737 | 0.0938 | Inf | 3.986 | **0.0002** |
| Control _B | Salinity_B |  | 0.026 | 0.0846 | Inf | 0.307 | 0.8393 |
| Control_B | CuSO_4__B |  | 0.5478 | 0.0964 | Inf | 5.685 | **<.0001** |
| Control_B | Both_B |  | 0.7783 | 0.104 | Inf | 7.486 | **<.0001** |
| CuSO_4__B | Salinity_B |  | -0.5218 | 0.0982 | Inf | -5.314 | **<.0001** |
| CuSO_4__B | Both_B |  | -0.1217 | 0.1053 | Inf | -1.156 | 0.4102 |
| Salinity _B | Both_B |  | 0.7523 | 0.1057 | Inf | 7.119 | **<.0001** |
| Control_C | Salinity_C |  | 0 | 0.084 | Inf | 0 | 1 |
| Control_C | CuSO_4__C |  | 0.4004 | 0.0921 | Inf | 4.348 | **<.0001** |
| Control_C | Both_C |  | 0.5419 | 0.0962 | Inf | 5.635 | **<.0001** |
| CuSO_4__C | Salinity_C |  | -0.4004 | 0.0935 | Inf | -4.283 | **0.0001** |
| CuSO_4__C | Both_C |  | 0.1415 | 0.1045 | Inf | 1.354 | 0.3011 |
| Salinity _C | Both_C |  | 0.5419 | 0.0975 | Inf | 5.557 | **<.0001** |
| Control_D | Salinity_D |  | 0.011 | 0.0855 | Inf | 0.128 | 0.9376 |
| Control_D | CuSO_4__D |  | 1.7456 | 0.1564 | Inf | 11.159 | **<.0001** |
| Control_D | Both_D |  | 1.0629 | 0.119 | Inf | 8.931 | **<.0001** |
| CuSO_4__D | Salinity_D |  | -1.7346 | 0.1566 | Inf | -11.08 | **<.0001** |
| CuSO_4__D | Both_D |  | -0.6827 | 0.1771 | Inf | -3.855 | **0.0003** |
| Salinity_D | Both_D |  | 1.0519 | 0.1192 | Inf | 8.827 | **<.0001** |
| Control_E | Salinity_E |  | 0.0361 | 0.0873 | Inf | 0.413 | 0.7954 |
| Control_E | CuSO_4__E |  | 2.1013 | 0.1817 | Inf | 11.566 | **<.0001** |
| Control_E | Both_E |  | 0.8318 | 0.1089 | Inf | 7.638 | **<.0001** |
| CuSO_4__E | Salinity_E |  | -2.0652 | 0.1828 | Inf | -11.296 | **<.0001** |
| CuSO_4__E | Both_E |  | -1.2694 | 0.1941 | Inf | -6.54 | **<.0001** |
| Salinity_E | Both_E |  | 0.7958 | 0.1108 | Inf | 7.181 | **<.0001** |
| Control_F | Salinity_F |  | 0.1204 | 0.0869 | Inf | 1.387 | 0.289 |
| Control_F | CuSO_4__F |  | 1.5644 | 0.1432 | Inf | 10.927 | **<.0001** |
| Control_F | Both_F |  | 1.131 | 0.1206 | Inf | 9.381 | **<.0001** |
| CuSO_4__F | Salinity_F |  | -1.4439 | 0.1447 | Inf | -9.976 | **<.0001** |
| CuSO_4__F | Both_F |  | -0.4333 | 0.1671 | Inf | -2.592 | 0.0203 |
| Salinity_F | Both_F |  | 1.0106 | 0.1224 | Inf | 8.255 | **<.0001** |
| Control_A | Control_B |  | 0 | 0.0825 | Inf | 0 | 1 |
| Control_A | Control_C |  | 0 | 0.0825 | Inf | 0 | 1 |
| Control_A | Control_D |  | 0.0668 | 0.0839 | Inf | 0.796 | 0.5924 |
| Control_A | Control_E |  | 0.056 | 0.0837 | Inf | 0.669 | 0.6533 |
| Control_A | Control_F |  | 0.0417 | 0.0834 | Inf | 0.5 | 0.7405 |
| Control_B | Control_C |  | 0 | 0.0825 | Inf | 0 | 1 |
| Control_B | Control_D |  | 0.0668 | 0.0839 | Inf | 0.796 | 0.5924 |
| Control_B | Control_E |  | 0.056 | 0.0837 | Inf | 0.669 | 0.6533 |
| Control_B | Control_F |  | 0.0417 | 0.0834 | Inf | 0.5 | 0.7405 |
| Control_C | Control_D |  | 0.0668 | 0.0839 | Inf | 0.796 | 0.5924 |
| Control_C | Control_E |  | 0.056 | 0.0837 | Inf | 0.669 | 0.6533 |
| Control_C | Control_F |  | 0.0417 | 0.0834 | Inf | 0.5 | 0.7405 |
| Control_D | Control_E |  | -0.0108 | 0.085 | Inf | -0.128 | 0.9376 |
| Control_D | Control_F |  | -0.0251 | 0.0847 | Inf | -0.297 | 0.8393 |
| Control_E | Control_F |  | -0.0143 | 0.0845 | Inf | -0.169 | 0.9291 |
| Salinity_A | Salinity_B |  | -0.0264 | 0.0857 | Inf | -0.308 | 0.8393 |
| Salinity_A | Salinity_C |  | -0.0524 | 0.0851 | Inf | -0.615 | 0.6802 |
| Salinity_A | Salinity_D |  | 0.0254 | 0.0852 | Inf | 0.298 | 0.8393 |
| Salinity_A | Salinity_E |  | 0.0397 | 0.0872 | Inf | 0.455 | 0.7695 |
| Salinity_A | Salinity_F |  | 0.1098 | 0.0871 | Inf | 1.26 | 0.3496 |
| Salinity_B | Salinity_C |  | -0.026 | 0.0862 | Inf | -0.302 | 0.8393 |
| Salinity_B | Salinity_D |  | 0.0518 | 0.0862 | Inf | 0.601 | 0.6832 |
| Salinity_B | Salinity_E |  | 0.066 | 0.0882 | Inf | 0.749 | 0.6137 |
| Salinity_B | Salinity_F |  | 0.1361 | 0.0881 | Inf | 1.546 | 0.2245 |
| Salinity_C | Salinity_D |  | 0.0778 | 0.0857 | Inf | 0.908 | 0.5459 |
| Salinity_C | Salinity_E |  | 0.092 | 0.0876 | Inf | 1.05 | 0.4547 |
| Salinity_C | Salinity_F |  | 0.1621 | 0.0875 | Inf | 1.852 | 0.1229 |
| Salinity_D | Salinity_E |  | 0.0142 | 0.0877 | Inf | 0.162 | 0.9291 |
| Salinity_D | Salinity_F |  | 0.0843 | 0.0876 | Inf | 0.963 | 0.5116 |
| Salinity_E | Salinity_F |  | 0.0701 | 0.0895 | Inf | 0.783 | 0.5947 |
| CuSO_4__A | CuSO_4__B |  | 0.2348 | 0.1026 | Inf | 2.288 | 0.0452 |
| CuSO_4__A | CuSO_4__C |  | 0.0874 | 0.0986 | Inf | 0.887 | 0.5543 |
| CuSO_4__A | CuSO_4__D |  | 1.4994 | 0.1596 | Inf | 9.393 | **<.0001** |
| CuSO_4__A | CuSO_4__E |  | 1.8443 | 0.1846 | Inf | 9.993 | **<.0001** |
| CuSO_4__A | CuSO_4__F |  | 1.2931 | 0.147 | Inf | 8.798 | **<.0001** |
| CuSO_4__B | CuSO_4__C |  | -0.1474 | 0.1047 | Inf | -1.408 | 0.2828 |
| CuSO_4__B | CuSO_4__D |  | 1.2646 | 0.1634 | Inf | 7.737 | **<.0001** |
| CuSO_4__B | CuSO_4__E |  | 1.6094 | 0.1879 | Inf | 8.567 | **<.0001** |
| CuSO_4__B | CuSO_4__F |  | 1.0583 | 0.1511 | Inf | 7.004 | **<.0001** |
| CuSO_4__C | CuSO_4__D |  | 1.412 | 0.161 | Inf | 8.772 | **<.0001** |
| CuSO_4__C | CuSO_4__E |  | 1.7568 | 0.1857 | Inf | 9.46 | **<.0001** |
| CuSO_4__C | CuSO_4__F |  | 1.2057 | 0.1484 | Inf | 8.124 | **<.0001** |
| CuSO_4__D | CuSO_4__E |  | 0.3448 | 0.2242 | Inf | 1.538 | 0.2245 |
| CuSO_4__D | CuSO_4__F |  | -0.2063 | 0.1944 | Inf | -1.062 | 0.454 |
| CuSO_4__E | CuSO_4__F |  | -0.5512 | 0.2153 | Inf | -2.56 | **0.0219** |
| Both_A | Both_B |  | 0.3522 | 0.1123 | Inf | 3.136 | **0.0041** |
| Both_A | Both_C |  | 0.1158 | 0.1051 | Inf | 1.102 | 0.433 |
| Both_A | Both_D |  | 0.7036 | 0.1254 | Inf | 5.609 | **<.0001** |
| Both_A | Both_E |  | 0.4617 | 0.1161 | Inf | 3.978 | **0.0002** |
| Both_A | Both_F |  | 0.7466 | 0.1273 | Inf | 5.867 | **<.0001** |
| Both_B | Both_C |  | -0.2364 | 0.1151 | Inf | -2.053 | 0.0785 |
| Both_B | Both_D |  | 0.3514 | 0.1339 | Inf | 2.624 | **0.019** |
| Both_B | Both_E |  | 0.1095 | 0.1252 | Inf | 0.875 | 0.5554 |
| Both_B | Both_F |  | 0.3944 | 0.1356 | Inf | 2.908 | **0.0083** |
| Both_C | Both_D |  | 0.5878 | 0.128 | Inf | 4.593 | **<.0001** |
| Both_C | Both_E |  | 0.3459 | 0.1188 | Inf | 2.911 | **0.0083** |
| Both_C | Both_F |  | 0.6308 | 0.1298 | Inf | 4.861 | **<.0001** |
| Both_D | Both_E |  | -0.2419 | 0.1371 | Inf | -1.765 | 0.1461 |
| Both_D | Both_F |  | 0.043 | 0.1467 | Inf | 0.293 | 0.8393 |
| Both_E | Both_F |  | 0.2849 | 0.1388 | Inf | 2.053 | 0.0785 |

Table S3: Complete Summary of Generalized Linear Model Analysis – Effects of Genotype, CuSO₄, and Salinity on *Daphnia magna* survival. Results include estimates, effect sizes (Incident Rate Ratio, IRR), standard errors, z-values, and p-values.

|  | Estimate | IRR | Standard error | z value | P-value |
| --- | --- | --- | --- | --- | --- |
| (Intercept) | 3.0445 | 21.00 | 0.0583 | 52.2027 | **<2E-16** |
| Genotype B | 5.616E-15 | 1.00 | 0.0825 | 0.00 | 1.00 |
| Genotype C | 4.122E-15 | 1.00 | 0.0825 | 0.00 | 1.00 |
| Genotype D | -0.0668 | 0.9354 | 0.0839 | -0.7964 | 0.4258 |
| Genotype E | -0.0560 | 0.9456 | 0.0837 | -0.6689 | 0.5036 |
| Genotype F | -0.0417 | 0.9592 | 0.0834 | -0.5000 | 0.6171 |
| Salinity | -0.0524 | 0.9490 | 0.0836 | -0.6266 | 0.5309 |
| CuSO_4_ | -0.3129 | 0.7313 | 0.0897 | -3.4874 | **0.0005** |
| Genotype B:Salinity | 0.0264 | 1.0267 | 0.1189 | 0.2219 | 0.8244 |
| Genotype C:Salinity | 0.0524 | 1.0538 | 0.1185 | 0.4418 | 0.6586 |
| Genotype D:Salinity | 0.0414 | 1.0423 | 0.1196 | 0.3462 | 0.7292 |
| Genotype E:Salinity | 0.0163 | 1.0164 | 0.1208 | 0.1350 | 0.8926 |
| Genotype F:Salinity | -0.0681 | 0.9342 | 0.1205 | -0.5647 | 0.5722 |
| Genotype B:CuSO_4_ | -0.2348 | 0.7907 | 0.1317 | -1.7836 | 0.0745 |
| Genotype C:CuSO_4_ | -0.0874 | 0.9163 | 0.1286 | -0.6801 | 0.4965 |
| Genotype D:CuSO_4_ | -1.4326 | 0.2387 | 0.1803 | -7.9441 | **1.96E-15** |
| Genotype E:CuSO_4_ | -1.7883 | 0.1672 | 0.2026 | -8.8253 | **<2E-16** |
| Genotype F:CuSO_4_ | -1.2514 | 0.2861 | 0.1690 | -7.4066 | **1.30E-13** |
| Salinity:CuSO_4_ | -0.0608 | 0.9410 | 0.1298 | -0.4683 | 0.6396 |
| Genotype B:Salinity:CuSO_4_ | -0.1438 | 0.8661 | 0.1931 | -0.7445 | 0.4566 |
| Genotype C:Salinity:CuSO_4_ | -0.0808 | 0.9224 | 0.1866 | -0.4327 | 0.6652 |
| Genotype D:Salinity:CuSO_4_ | 0.7544 | 2.1264 | 0.2356 | 3.2018 | **0.0014** |
| Genotype E:Salinity:CuSO_4_ | 1.3663 | 3.9207 | 0.2493 | 5.4811 | **4.23E-08** |
| Genotype F:Salinity:CuSO_4_ | 0.6145 | 1.8488 | 0.2288 | 2.6865 | **0.0072** |

Table S4: Mean and standard error of days alive as an indicator of survival in *Daphnia magna* across all experimental conditions.

| Genotype | Location | Treatment | Mean | Standard error |
| --- | --- | --- | --- | --- |
| A | France | Control | 21.0 | 0.0 |
| A | France | CuSO_4_ | 15.4 | 1.43 |
| A | France | Elevated salinity | 19.9 | 0.774 |
| A | France | Elevated salinityx CuSO_4_ | 13.7 | 0.21 |
| B | France | Control | 21.0 | 0.0 |
| B | France | CuSO_4_ | 12.1 | 1.17 |
| B | France | Elevated salinity | 20.5 | 0.538 |
| B | France | Elevated salinityx CuSO_4_ | 9.64 | 1.06 |
| C | France | Control | 21.0 | 0.0 |
| C | France | CuSO_4_ | 14.1 | 1.74 |
| C | France | Elevated salinity | 21.0 | 0.0 |
| C | France | Elevated salinityx CuSO_4_ | 12.2 | 1.04 |
| D | USA | Control | 19.6 | 1.36 |
| D | USA | CuSO_4_ | 3.43 | 0.477 |
| D | USA | Elevated salinity | 19.4 | 1.24 |
| D | USA | Elevated salinityx CuSO_4_ | 6.79 | 1.78 |
| E | USA | Control | 19.9 | 0.523 |
| E | USA | CuSO_4_ | 2.43 | 0.228 |
| E | USA | Elevated salinity | 19.2 | 0.846 |
| E | USA | Elevated salinityx CuSO_4_ | 8.64 | 1.17 |
| F | USA | Control | 20.1 | 0.592 |
| F | USA | CuSO_4_ | 4.21 | 0.689 |
| F | USA | Elevated salinity | 17.9 | 1.57 |
| F | USA | Elevated salinityx CuSO_4_ | 6.5 | 0.717 |

Table S5: Custom Contrast Analysis comparing *Daphnia magna* total fecundity across various combinations of elevated salinity and CuSO_4_ among multiple genotypes from two distinct location. Results are shown for comparisons amongst and between controls, elevated salinity (“Salinity”), CuSO_4_, and both stressors combined (“Both”) across all genotypes (A to F). Significant differences are highlighted in bold.

| **Contrast** (Treatment_Genotype) | | | **Estimate** | **SE** | **df** | **z ratio** | **p-value** |
| --- | --- | --- | --- | --- | --- | --- | --- |
| **Group 1** | **Group 2** |  |  |  |  |  |  |
| Control_A | Salinity_A |  | 0.7970 | 0.293 | Inf | 2.721 | **0.0201** |
| Control_A | CuSO_4__A |  | 4.1352 | 0.497 | Inf | 8.328 | **<.0001** |
| Control _A | Both_A |  | 3.442 | 0.404 | Inf | 8.521 | **<.0001** |
| CuSO_4__A | Salinity_A |  | -3.3381 | 0.5 | Inf | -6.679 | **<.0001** |
| CuSO_4__A | Both_A |  | -0.6931 | 0.572 | Inf | -1.212 | 0.4708 |
| Salinity_A | Both_A |  | 2.645 | 0.408 | Inf | 6.483 | **<.0001** |
| Control _B | Salinity_B |  | 1.6064 | 0.309 | Inf | 5.194 | **<.0001** |
| Control_B | CuSO_4__B |  | 4.3944 | 0.575 | Inf | 7.647 | **<.0001** |
| Control_B | Both_B |  | 24.1374 | 5874.272 | Inf | 0.004 | 1 |
| CuSO_4__B | Salinity_B |  | -2.7881 | 0.585 | Inf | -4.764 | **<.0001** |
| CuSO_4__B | Both_B |  | -1.0986 | 0.641 | Inf | -1.715 | 0.2074 |
| Salinity _B | Both_B |  | 22.5311 | 5874.272 | Inf | 0.004 | 1 |
| Control_C | Salinity_C |  | 0.1156 | 0.289 | Inf | 0.401 | 1 |
| Control_C | CuSO_4__C |  | 3.1442 | 0.383 | Inf | 8.208 | **<.0001** |
| Control_C | Both_C |  | 4.2428 | 0.529 | Inf | 8.017 | **<.0001** |
| CuSO_4__C | Salinity_C |  | -3.0285 | 0.384 | Inf | -7.897 | **<.0001** |
| CuSO_4__C | Both_C |  | 1.0986 | 0.586 | Inf | 1.874 | 0.1541 |
| Salinity _C | Both_C |  | 4.1271 | 0.53 | Inf | 7.794 | **<.0001** |
| Control_D | Salinity_D |  | 1.6829 | 0.325 | Inf | 5.183 | **<.0001** |
| Control_D | CuSO_4__D |  | 23.7772 | 5874.272 | Inf | 0.004 | 1 |
| Control_D | Both_D |  | 23.7772 | 5874.272 | Inf | 0.004 | 1 |
| CuSO_4__D | Salinity_D |  | -22.0943 | 5874.272 | Inf | -0.004 | 1 |
| CuSO_4__D | Both_D |  | 0 | 8307.476 | Inf | 0 | 1 |
| Salinity_D | Both_D |  | 22.0943 | 5874.272 | Inf | 0.004 | 1 |
| Control_E | Salinity_E |  | 1.0609 | 0.476 | Inf | 2.228 | 0.0753 |
| Control_E | CuSO_4__E |  | 21.6148 | 5874.272 | Inf | 0.004 | 1 |
| Control_E | Both_E |  | 21.6148 | 5874.272 | Inf | 0.004 | 1 |
| CuSO_4__E | Salinity_E |  | -20.5539 | 5874.272 | Inf | -0.003 | 1 |
| CuSO_4__E | Both_E |  | 0 | 8307.476 | Inf | 0 | 1 |
| Salinity_E | Both_E |  | 20.5539 | 5874.272 | Inf | 0.003 | 1 |
| Control_F | Salinity_F |  | 3.4286 | 0.499 | Inf | 6.867 | **<.0001** |
| Control_F | CuSO_4__F |  | 23.577 | 5874.272 | Inf | 0.004 | 1 |
| Control_F | Both_F |  | 23.577 | 5874.272 | Inf | 0.004 | 1 |
| CuSO_4__F | Salinity_F |  | -20.1484 | 5874.272 | Inf | -0.003 | 1 |
| CuSO_4__F | Both_F |  | 0 | 8307.476 | Inf | 0 | 1 |
| Salinity_F | Both_F |  | 20.1484 | 5874.272 | Inf | 0.003 | 1 |
| Control_A | Control_B |  | 0.1462 | 0.288 | Inf | 0.508 | 1 |
| Control_A | Control_C |  | 0.0747 | 0.288 | Inf | 0.26 | 1 |
| Control_A | Control_D |  | 0.5064 | 0.29 | Inf | 1.744 | 0.1997 |
| Control_A | Control_E |  | 2.6688 | 0.344 | Inf | 7.759 | **<.0001** |
| Control_A | Control_F |  | 0.7066 | 0.292 | Inf | 2.42 | **0.0466** |
| Control_B | Control_C |  | -0.0715 | 0.288 | Inf | -0.248 | 1 |
| Control_B | Control_D |  | 0.3602 | 0.291 | Inf | 1.238 | 0.4604 |
| Control_B | Control_E |  | 2.5226 | 0.345 | Inf | 7.321 | **<.0001** |
| Control_B | Control_F |  | 0.5604 | 0.293 | Inf | 1.914 | 0.1442 |
| Control_C | Control_D |  | 0.4317 | 0.291 | Inf | 1.485 | 0.3143 |
| Control_C | Control_E |  | 2.5941 | 0.344 | Inf | 7.535 | **<.0001** |
| Control_C | Control_F |  | 0.6318 | 0.292 | Inf | 2.161 | 0.0866 |
| Control_D | Control_E |  | 2.1624 | 0.347 | Inf | 6.24 | **<.0001** |
| Control_D | Control_F |  | 0.2002 | 0.295 | Inf | 0.679 | 0.9948 |
| Control_E | Control_F |  | -1.9623 | 0.348 | Inf | -5.64 | **<.0001** |
| Salinity_A | Salinity_B |  | 0.9555 | 0.314 | Inf | 3.045 | **0.0075** |
| Salinity_A | Salinity_C |  | -0.6067 | 0.294 | Inf | -2.065 | 0.1039 |
| Salinity_A | Salinity_D |  | 1.3922 | 0.327 | Inf | 4.258 | **0.0001** |
| Salinity_A | Salinity_E |  | 2.9327 | 0.441 | Inf | 6.655 | **<.0001** |
| Salinity_A | Salinity_F |  | 3.3381 | 0.5 | Inf | 6.679 | **<.0001** |
| Salinity_B | Salinity_C |  | -1.5622 | 0.31 | Inf | -5.047 | **<.0001** |
| Salinity_B | Salinity_D |  | 0.4367 | 0.341 | Inf | 1.28 | 0.4375 |
| Salinity_B | Salinity_E |  | 1.9772 | 0.451 | Inf | 4.381 | **<.0001** |
| Salinity_B | Salinity_F |  | 2.3826 | 0.509 | Inf | 4.68 | **<.0001** |
| Salinity_C | Salinity_D |  | 1.9989 | 0.323 | Inf | 6.192 | **<.0001** |
| Salinity_C | Salinity_E |  | 3.5393 | 0.438 | Inf | 8.087 | **<.0001** |
| Salinity_C | Salinity_F |  | 3.9448 | 0.497 | Inf | 7.936 | **<.0001** |
| Salinity_D | Salinity_E |  | 1.5404 | 0.461 | Inf | 3.345 | **0.0027** |
| Salinity_D | Salinity_F |  | 1.9459 | 0.517 | Inf | 3.761 | **0.0006** |
| Salinity_E | Salinity_F |  | 0.4055 | 0.596 | Inf | 0.681 | 0.9948 |
| CuSO_4__A | CuSO_4__B |  | 0.4055 | 0.703 | Inf | 0.577 | 1 |
| CuSO_4__A | CuSO_4__C |  | -0.9163 | 0.557 | Inf | -1.644 | 0.2344 |
| CuSO_4__A | CuSO_4__D |  | 20.1484 | 5874.272 | Inf | 0.003 | 1 |
| CuSO_4__A | CuSO_4__E |  | 20.1484 | 5874.272 | Inf | 0.003 | 1 |
| CuSO_4__A | CuSO_4__F |  | 20.1484 | 5874.272 | Inf | 0.003 | 1 |
| CuSO_4__B | CuSO_4__C |  | -1.3218 | 0.628 | Inf | -2.106 | 0.0965 |
| CuSO_4__B | CuSO_4__D |  | 19.743 | 5874.272 | Inf | 0.003 | 1 |
| CuSO_4__B | CuSO_4__E |  | 19.743 | 5874.272 | Inf | 0.003 | 1 |
| CuSO_4__B | CuSO_4__F |  | 19.743 | 5874.272 | Inf | 0.003 | 1 |
| CuSO_4__C | CuSO_4__D |  | 21.0647 | 5874.272 | Inf | 0.004 | 1 |
| CuSO_4__C | CuSO_4__E |  | 21.0647 | 5874.272 | Inf | 0.004 | 1 |
| CuSO_4__C | CuSO_4__F |  | 21.0647 | 5874.272 | Inf | 0.004 | 1 |
| CuSO_4__D | CuSO_4__E |  | 0 | 8307.476 | Inf | 0 | 1 |
| CuSO_4__D | CuSO_4__F |  | 0 | 8307.476 | Inf | 0 | 1 |
| CuSO_4__E | CuSO_4__F |  | 0 | 8307.476 | Inf | 0 | 1 |
| Both_A | Both_B |  | 20.8416 | 5874.272 | Inf | 0.004 | 1 |
| Both_A | Both_C |  | 0.8755 | 0.6 | Inf | 1.458 | 0.3233 |
| Both_A | Both_D |  | 20.8416 | 5874.272 | Inf | 0.004 | 1 |
| Both_A | Both_E |  | 20.8416 | 5874.272 | Inf | 0.004 | 1 |
| Both_A | Both_F |  | 20.8416 | 5874.272 | Inf | 0.004 | 1 |
| Both_B | Both_C |  | -19.966 | 5874.272 | Inf | -0.003 | 1 |
| Both_B | Both_D |  | 0 | 8307.476 | Inf | 0 | 1 |
| Both_B | Both_E |  | 0 | 8307.476 | Inf | 0 | 1 |
| Both_B | Both_F |  | 0 | 8307.476 | Inf | 0 | 1 |
| Both_C | Both_D |  | 19.9661 | 5874.272 | Inf | 0.003 | 1 |
| Both_C | Both_E |  | 19.9661 | 5874.272 | Inf | 0.003 | 1 |
| Both_C | Both_F |  | 19.9661 | 5874.272 | Inf | 0.003 | 1 |
| Both_D | Both_E |  | 0 | 8307.476 | Inf | 0 | 1 |
| Both_D | Both_F |  | 0 | 8307.476 | Inf | 0 | 1 |
| Both_E | Both_F |  | 0 | 8307.476 | Inf | 0 | 1 |

Table S6: Summary of Generalized Linear Model analysis: Effects of Genotype, CuSO_4_, and Salinity on the offspring female-to-male ratio in *D. magna*. Results are shown for Degrees of Freedom (Df), Deviance, Residual Df, Residual deviance and P-Values.

|  | **Df** | **Deviance** | **Residual Df** | **Residual deviance** | **P-Value** |  |
| --- | --- | --- | --- | --- | --- | --- |
| NULL |  |  | 95 | 453.04 |  |  |
| Genotype | 5 | 105.298 | 90 | 347.74 | **<2.16E-16** | ******* |
| Salinity | 1 | 104.920 | 89 | 242.82 | **<2.16E-16** | *** |
| CuSO_4_ | 1 | 150.313 | 88 | 92.51 | **<2.16E-16** | ******* |
| Genotype:Salinity | 5 | 15.867 | 83 | 76.64 | **0.0072** | ** |
| Genotype:CuSO_4_ | 5 | 6.941 | 78 | 69.70 | 0.2251 |  |
| Salinity:CuSO_4_ | 1 | 1.599 | 77 | 68.10 | 0.2061 |  |
| Genotype:Salinity :CuSO_4_ | 5 | 2.367 | 72 | 65.74 | 0.7964 |  |

Table S7: Custom Contrast Analysis comparing *Daphnia magna* offspring female-to-male ratio across various combinations of elevated salinity and CuSO_4_ among multiple genotypes from two distinct location. Results are shown for comparisons amongst and between controls, elevated salinity (“Salinity”), CuSO_4_, and both stressors combined (“Both”) across all genotypes (A to F). Significant differences are highlighted in bold.

| **Contrast** (Treatment_Genotype) | | | **Estimate** | **SE** | **df** | **z ratio** | **p-value** |
| --- | --- | --- | --- | --- | --- | --- | --- |
| **Group 1** | **Group 2** |  |  |  |  |  |  |
| Control_A | Salinity_A |  | 2.5535 | 6.22e-01 | Inf | 4.107 | **0.0004** |
| Control_A | CuSO_4__A |  | 4.8727 | 1.14e+00 | Inf | 4.293 | **0.0002** |
| Control _A | Both_A |  | 4.5362 | 1.00e+00 | Inf | 4.530 | **0.0001** |
| CuSO_4__A | Salinity_A |  | -2.3191 | 1.17e+00 | Inf | -1.975 | 0.1931 |
| CuSO_4__A | Both_A |  | -0.3365 | 1.41e+00 | Inf | -0.238 | 1.0000 |
| Salinity_A | Both_A |  | 1.9826 | 1.05e+00 | Inf | 1.896 | 0.2225 |
| Control _B | Salinity_B |  | 2.9982 | 6.75e-01 | Inf | 4.442 | **0.0001** |
| Control_B | CuSO_4__B |  | 3.6914 | 7.89e-01 | Inf | 4.680 | **<.0001** |
| Control_B | Both_B |  | 23.7063 | 7.77e+03 | Inf | 0.003 | 1.0000 |
| CuSO_4__B | Salinity_B |  | -0.6931 | 8.84e-01 | Inf | -0.785 | 1.0000 |
| CuSO_4__B | Both_B |  | 0.7621 | 1.15e+00 | Inf | 0.661 | 1.0000 |
| Salinity _B | Both_B |  | 20.7081 | 7.77e+03 | Inf | 0.003 | 1.0000 |
| Control_C | Salinity_C |  | 0.9779 | 5.54e-01 | Inf | 1.766 | 0.2752 |
| Control_C | CuSO_4__C |  | 3.1534 | 6.74e-01 | Inf | 4.678 | **<.0001** |
| Control_C | Both_C |  | 3.5589 | 7.33e-01 | Inf | 4.853 | **<.0001** |
| CuSO_4__C | Salinity_C |  | -2.1755 | 6.83e-01 | Inf | -3.186 | **0.0081** |
| CuSO_4__C | Both_C |  | 0.4055 | 8.35e-01 | Inf | 0.486 | 1.0000 |
| Salinity _C | Both_C |  | 2.5810 | 7.41e-01 | Inf | 3.482 | **0.0034** |
| Control_D | Salinity_D |  | 3.6058 | 9.36e-01 | Inf | 3.850 | **0.0009** |
| Control_D | CuSO_4__D |  | 23.0658 | 7.77e+03 | Inf | 0.003 | 1.0000 |
| Control_D | Both_D |  | 23.0658 | 7.77e+03 | Inf | 0.003 | 1.0000 |
| CuSO_4__D | Salinity_D |  | -19.4599 | 7.77e+03 | Inf | -0.003 | 1.0000 |
| CuSO_4__D | Both_D |  | 0.0000 | 1.10e+04 | Inf | 0.000 | 1.0000 |
| Salinity_D | Both_D |  | 19.4599 | 7.77e+03 | Inf | 0.003 | 1.0000 |
| Control_E | Salinity_E |  | 19.9279 | 7.77e+03 | Inf | 0.003 | 1.0000 |
| Control_E | CuSO_4__E |  | 19.9279 | 7.77e+03 | Inf | 0.003 | 1.0000 |
| Control_E | Both_E |  | 19.9279 | 7.77e+03 | Inf | 0.003 | 1.0000 |
| CuSO_4__E | Salinity_E |  | 0.0000 | 1.10e+04 | Inf | 0.000 | 1.0000 |
| CuSO_4__E | Both_E |  | 0.0000 | 1.10e+04 | Inf | 0.000 | 1.0000 |
| Salinity_E | Both_E |  | 0.0000 | 1.10e+04 | Inf | 0.000 | 1.0000 |
| Control_F | Salinity_F |  | 5.2709 | 2.30e+00 | Inf | 2.288 | 0.1012 |
| Control_F | CuSO_4__F |  | 22.5777 | 7.77e+03 | Inf | 0.003 | 1.0000 |
| Control_F | Both_F |  | 22.5777 | 7.77e+03 | Inf | 0.003 | 1.0000 |
| CuSO_4__F | Salinity_F |  | -17.3069 | 7.77e+03 | Inf | -0.002 | 1.0000 |
| CuSO_4__F | Both_F |  | 0.0000 | 1.10e+04 | Inf | 0.000 | 1.0000 |
| Salinity_F | Both_F |  | 17.3069 | 7.77e+03 | Inf | 0.002 | 1.0000 |
| Control_A | Control_B |  | 0.0827 | 5.45e-01 | Inf | 0.152 | 1.0000 |
| Control_A | Control_C |  | -0.0726 | 5.43e-01 | Inf | -0.134 | 1.0000 |
| Control_A | Control_D |  | 0.7232 | 5.51e-01 | Inf | 1.312 | 0.5689 |
| Control_A | Control_E |  | 3.8610 | 8.07e-01 | Inf | 4.782 | **<.0001** |
| Control_A | Control_F |  | 1.2112 | 5.60e-01 | Inf | 2.162 | 0.1337 |
| Control_B | Control_C |  | -0.1552 | 5.44e-01 | Inf | -0.285 | 1.0000 |
| Control_B | Control_D |  | 0.6405 | 5.52e-01 | Inf | 1.160 | 0.6942 |
| Control_B | Control_E |  | 3.7784 | 8.08e-01 | Inf | 4.677 | **<.0001** |
| Control_B | Control_F |  | 1.1286 | 5.61e-01 | Inf | 2.012 | 0.1845 |
| Control_C | Control_D |  | 0.7957 | 5.51e-01 | Inf | 1.445 | 0.4602 |
| Control_C | Control_E |  | 3.9336 | 8.07e-01 | Inf | 4.874 | **<.0001** |
| Control_C | Control_F |  | 1.2838 | 5.60e-01 | Inf | 2.293 | 0.1012 |
| Control_D | Control_E |  | 3.1379 | 8.12e-01 | Inf | 3.862 | **0.0009** |
| Control_D | Control_F |  | 0.4880 | 5.67e-01 | Inf | 0.860 | 0.9847 |
| Control_E | Control_F |  | -2.6498 | 8.18e-01 | Inf | -3.238 | **0.0077** |
| Salinity_A | Salinity_B |  | 0.5274 | 7.39e-01 | Inf | 0.714 | 1.0000 |
| Salinity_A | Salinity_C |  | -1.6482 | 6.31e-01 | Inf | -2.613 | **0.0479** |
| Salinity_A | Salinity_D |  | 1.7755 | 9.80e-01 | Inf | 1.813 | 0.2581 |
| Salinity_A | Salinity_E |  | 21.2354 | 7.77e+03 | Inf | 0.003 | 1.0000 |
| Salinity_A | Salinity_F |  | 3.9286 | 2.32e+00 | Inf | 1.694 | 0.3096 |
| Salinity_B | Salinity_C |  | -2.1755 | 6.83e-01 | Inf | -3.186 | **0.0081** |
| Salinity_B | Salinity_D |  | 1.2481 | 1.01e+00 | Inf | 1.231 | 0.6350 |
| Salinity_B | Salinity_E |  | 20.7081 | 7.77e+03 | Inf | 0.003 | 1.0000 |
| Salinity_B | Salinity_F |  | 3.4012 | 2.33e+00 | Inf | 1.457 | 0.4602 |
| Salinity_C | Salinity_D |  | 3.4237 | 9.38e-01 | Inf | 3.649 | **0.0019** |
| Salinity_C | Salinity_E |  | 22.8836 | 7.77e+03 | Inf | 0.003 | 1.0000 |
| Salinity_C | Salinity_F |  | 5.5767 | 2.30e+00 | Inf | 2.422 | 0.0779 |
| Salinity_D | Salinity_E |  | 19.4599 | 7.77e+03 | Inf | 0.003 | 1.0000 |
| Salinity_D | Salinity_F |  | 2.1531 | 2.42e+00 | Inf | 0.889 | 0.9847 |
| Salinity_E | Salinity_F |  | -17.3069 | 7.77e+03 | Inf | -0.002 | 1.0000 |
| CuSO_4__A | CuSO_4__B |  | -1.0986 | 1.27e+00 | Inf | -0.865 | 0.9847 |
| CuSO_4__A | CuSO_4__C |  | -1.7918 | 1.20e+00 | Inf | -1.489 | 0.4515 |
| CuSO_4__A | CuSO_4__D |  | 18.9163 | 7.77e+03 | Inf | 0.002 | 1.0000 |
| CuSO_4__A | CuSO_4__E |  | 18.9163 | 7.77e+03 | Inf | 0.002 | 1.0000 |
| CuSO_4__A | CuSO_4__F |  | 18.9163 | 7.77e+03 | Inf | 0.002 | 1.0000 |
| CuSO_4__B | CuSO_4__C |  | -0.6931 | 8.84e-01 | Inf | -0.785 | 1.0000 |
| CuSO_4__B | CuSO_4__D |  | 20.0149 | 7.77e+03 | Inf | 0.003 | 1.0000 |
| CuSO_4__B | CuSO_4__E |  | 20.0149 | 7.77e+03 | Inf | 0.003 | 1.0000 |
| CuSO_4__B | CuSO_4__F |  | 20.0149 | 7.77e+03 | Inf | 0.003 | 1.0000 |
| CuSO_4__C | CuSO_4__D |  | 20.7081 | 7.77e+03 | Inf | 0.003 | 1.0000 |
| CuSO_4__C | CuSO_4__E |  | 20.7081 | 7.77e+03 | Inf | 0.003 | 1.0000 |
| CuSO_4__C | CuSO_4__F |  | 20.7081 | 7.77e+03 | Inf | 0.003 | 1.0000 |
| CuSO_4__D | CuSO_4__E |  | 0.0000 | 1.10e+04 | Inf | 0.000 | 1.0000 |
| CuSO_4__D | CuSO_4__F |  | 0.0000 | 1.10e+04 | Inf | 0.000 | 1.0000 |
| CuSO_4__E | CuSO_4__F |  | 0.0000 | 1.10e+04 | Inf | 0.000 | 1.0000 |
| Both_A | Both_B |  | 19.2528 | 7.77e+03 | Inf | 0.002 | 1.0000 |
| Both_A | Both_C |  | -1.0498 | 1.12e+00 | Inf | -0.941 | 0.9511 |
| Both_A | Both_D |  | 19.2528 | 7.77e+03 | Inf | 0.002 | 1.0000 |
| Both_A | Both_E |  | 19.2528 | 7.77e+03 | Inf | 0.002 | 1.0000 |
| Both_A | Both_F |  | 19.2528 | 7.77e+03 | Inf | 0.002 | 1.0000 |
| Both_B | Both_C |  | -20.3026 | 7.77e+03 | Inf | -0.003 | 1.0000 |
| Both_B | Both_D |  | 0.0000 | 1.10e+04 | Inf | 0.000 | 1.0000 |
| Both_B | Both_E |  | 0.0000 | 1.10e+04 | Inf | 0.000 | 1.0000 |
| Both_B | Both_F |  | 0.0000 | 1.10e+04 | Inf | 0.000 | 1.0000 |
| Both_C | Both_D |  | 20.3026 | 7.77e+03 | Inf | 0.003 | 1.0000 |
| Both_C | Both_E |  | 20.3026 | 7.77e+03 | Inf | 0.003 | 1.0000 |
| Both_C | Both_F |  | 20.3026 | 7.77e+03 | Inf | 0.003 | 1.0000 |
| Both_D | Both_E |  | 0.0000 | 1.10e+04 | Inf | 0.000 | 1.0000 |
| Both_D | Both_F |  | 0.0000 | 1.10e+04 | Inf | 0.000 | 1.0000 |
| Both_E | Both_F |  | 0.0000 | 1.10e+04 | Inf | 0.000 | 1.0000 |

Table S8: Complete Summary of Generalized Linear Model Analysis – Effects of Genotype, CuSO₄, and Salinity on *Daphnia magna* total fecundity and female-to-male ratio. Results include estimates, effect sizes (Incident Rate Ratio, IRR), standard errors, z-values, and p-values.

| Total Fecundity |  |  |  |  |  |
| --- | --- | --- | --- | --- | --- |
|  | **Estimate** | **IRR** | **Standard error** | **z value** | **P-value** |
| (Intercept) | 3.9810 | 53.5714 | 0.2031 | 19.5984 | **<2.16E-16** |
| Genotype B | -0.1462 | 0.8640 | 0.2880 | -0.5076 | 0.6117 |
| Genotype C | -0.0747 | 0.9280 | 0.2876 | -0.2598 | 0.7950 |
| Genotype D | -0.5064 | 0.6027 | 0.2903 | -1.7443 | 0.0811 |
| Genotype E | -2.6688 | 0.0693 | 0.3440 | -7.7588 | **8.57E-15** |
| Genotype F | -0.7066 | 0.4933 | 0.2920 | -2.4198 | **0.0155** |
| Salinity | -0.7970 | 0.4507 | 0.2929 | -2.7214 | **0.0065** |
| CuSO_4_ | -4.1352 | 0.0160 | 0.4965 | -8.3284 | **<2.16E-16** |
| Genotype B:Salinity | -0.8093 | 0.4452 | 0.4260 | -1.9000 | 0.0574 |
| Genotype C:Salinity | 0.6814 | 1.9766 | 0.4112 | 1.6572 | 0.0975 |
| Genotype D:Salinity | -0.8858 | 0.4124 | 0.4373 | -2.0259 | **0.0428** |
| Genotype E:Salinity | -0.2638 | 0.7681 | 0.5591 | -0.4720 | 0.6370 |
| Genotype F:Salinity | -2.6316 | 0.0720 | 0.5788 | -4.5464 | **5.46E-06** |
| Genotype B:CuSO_4_ | -0.2593 | 0.7716 | 0.7595 | -0.3414 | 0.7328 |
| Genotype C:CuSO_4_ | 0.9910 | 2.6940 | 0.6271 | 1.5803 | 0.1140 |
| Genotype D:CuSO_4_ | -19.6420 | 0.0000 | 5874.2724 | -0.0033 | 0.9973 |
| Genotype E:CuSO_4_ | -17.4796 | 0.0000 | 5874.2724 | -0.0030 | 0.9976 |
| Genotype F:CuSO_4_ | -19.4419 | 0.0000 | 5874.2724 | -0.0033 | 0.9974 |
| Salinity:CuSO_4_ | 1.4902 | 4.4379 | 0.6426 | 2.3189 | **0.0204** |
| Genotype B:Salinity:CuSO_4_ | -19.6268 | 0.0000 | 5874.2724 | -0.0033 | 0.9973 |
| Genotype C:Salinity:CuSO_4_ | -2.4732 | 0.0843 | 0.9166 | -2.6983 | **0.0070** |
| Genotype D:Salinity:CuSO_4_ | 0.1927 | 1.2125 | 8307.4756 | 0.0000 | 1.0000 |
| Genotype E:Salinity:CuSO_4_ | -0.4293 | 0.6510 | 8307.4756 | -0.0001 | 1.0000 |
| Genotype F:Salinity:CuSO_4_ | 1.9384 | 6.9478 | 8307.4756 | 0.0002 | 0.9998 |
| Female-to-Male Ratio |  |  |  |  |  |
| (Intercept) | 3.4864 | 32.6667 | 0.3846 | 9.0642 | <2.16E-16 |
| Genotype B | -0.0827 | 0.9207 | 0.5446 | -0.1518 | 0.8793 |
| Genotype C | 0.0726 | 1.0753 | 0.5435 | 0.1335 | 0.8938 |
| Genotype D | -0.7232 | 0.4852 | 0.5514 | -1.3116 | 0.1896 |
| Genotype E | -3.8610 | 0.021 | 0.8074 | -4.7822 | 1.73E-06 |
| Genotype F | -1.2112 | 0.2978 | 0.5603 | -2.1618 | **0.0306** |
| Salinity | -2.5535 | 0.0778 | 0.6218 | -4.1069 | 4.01E-05 |
| CuSO_4_ | -4.8726 | 0.0077 | 1.1350 | -4.2931 | 1.76E-05 |
| Genotype B:Salinity | -0.4447 | 0.641 | 0.9177 | -0.4846 | 0.6280 |
| Genotype C:Salinity | 1.5756 | 4.8337 | 0.8326 | 1.8924 | 0.0584 |
| Genotype D:Salinity | -1.0523 | 0.3491 | 1.1241 | -0.9361 | 0.3492 |
| Genotype E:Salinity | -17.3744 | 0.0 | 7770.9319 | -0.0022 | 0.9982 |
| Genotype F:Salinity | -2.7173 | 0.0661 | 2.3860 | -1.1389 | 0.2548 |
| Genotype B:CuSO_4_ | 1.1813 | 3.2585 | 1.3822 | 0.8546 | 0.3928 |
| Genotype C:CuSO_4_ | 1.7192 | 5.5801 | 1.3201 | 1.3024 | 0.1928 |
| Genotype D:CuSO_4_ | -18.1931 | 0.0 | 7770.9320 | -0.0023 | 0.9981 |
| Genotype E:CuSO_4_ | -15.0552 | 0.0 | 7770.9320 | -0.0019 | 0.9985 |
| Genotype F:CuSO_4_ | -17.7051 | 0.0 | 7770.9320 | -0.0023 | 0.9982 |
| Salinity:CuSO_4_ | 2.8900 | 17.9934 | 1.5432 | 1.8727 | 0.0611 |
| Genotype B:Salinity:CuSO_4_ | -19.9067 | 0.0 | 7770.9321 | -0.0026 | 0.9980 |
| Genotype C:Salinity:CuSO_4_ | -2.3176 | 0.0985 | 1.8399 | -1.2596 | 0.2078 |
| Genotype D:Salinity:CuSO_4_ | 0.7158 | 2.0459 | 10989.7574 | 0.0001 | 0.9999 |
| Genotype E:Salinity:CuSO_4_ | 17.0379 | 2.50E+07 | 13459.6489 | 0.0013 | 0.9990 |
| Genotype F:Salinity:CuSO_4_ | 2.3809 | 10.8141 | 10989.7576 | 0.0002 | 0.9998 |

Table S9: Mean and standard error for total fecundity and the female-to-male ratio in *Daphnia magna* across all experimental conditions.

| Total Fecundity | |  |  |  |
| --- | --- | --- | --- | --- |
| Genotype | Location | Treatment | Mean | Standard error |
| A | France | Control | 53.6 | 6.44 |
| A | France | CuSO_4_ | 0.9 | 0.705 |
| A | France | Elevated salinity | 24.1 | 4.28 |
| A | France | Elevated salinity x CuSO_4_ | 1.7 | 1.71 |
| B | France | Control | 46.3 | 2.98 |
| B | France | CuSO_4_ | 0.6 | 0.571 |
| B | France | Elevated salinity | 9.3 | 3.56 |
| B | France | Elevated salinity x CuSO_4_ | 0.0 | 0.0 |
| C | France | Control | 49.7 | 2.97 |
| C | France | CuSO_4_ | 2.1 | 0.738 |
| C | France | Elevated salinity | 44.3 | 1.60 |
| C | France | Elevated salinity x CuSO_4_ | 0.7 | 0.714 |
| D | USA | Control | 32.3 | 2.95 |
| D | USA | CuSO_4_ | 0.0 | 0.0 |
| D | USA | Elevated salinity | 6.0 | 1.51 |
| D | USA | Elevated salinity x CuSO_4_ | 0.0 | 0.0 |
| E | USA | Control | 3.7 | 1.80 |
| E | USA | CuSO_4_ | 0.0 | 0.0 |
| E | USA | Elevated salinity | 1.3 | 0.714 |
| E | USA | Elevated salinity x CuSO_4_ | 0.0 | 0.0 |
| F | USA | Control | 26.4 | 1.77 |
| F | USA | CuSO_4_ | 0.0 | 0.0 |
| F | USA | Elevated salinity | 0.857 | 0.857 |
| F | USA | Elevated salinity x CuSO_4_ | 0.0 | 0.0 |
| Female-to-male ratio | |  |  |  |
| A | France | Control | 32,7 | 12,1 |
| A | France | CuSO_4_ | 0,3 | 0,25 |
| A | France | Elevated salinity | 2,5 | 1,07 |
| A | France | Elevated salinity x CuSO_4_ | 0,4 | 0,35 |
| B | France | Control | 30,1 | 8,36 |
| B | France | CuSO_4_ | 0,8 | 0,75 |
| B | France | Elevated salinity | 1,5 | 0,957 |
| B | France | Elevated salinity x CuSO_4_ | 0,0 | 0,0 |
| C | France | Control | 35,1 | 8,65 |
| C | France | CuSO_4_ | 1,5 | 0,866 |
| C | France | Elevated salinity | 13,2 | 10,9 |
| C | France | Elevated salinity x CuSO_4_ | 1,0 | 1,0 |
| D | USA | Control | 15,8 | 6,24 |
| D | USA | CuSO_4_ | 0,0 | 0 |
| D | USA | Elevated salinity | 4,31 | 0,216 |
| D | USA | Elevated salinity x CuSO_4_ | 0,0 | 0,0 |
| E | USA | Control | 0,7 | 0,425 |
| E | USA | CuSO_4_ | 0,0 | 0,0 |
| E | USA | Elevated salinity | 0,0 | 0,0 |
| E | USA | Elevated salinity x CuSO_4_ | 0,0 | 0,0 |
| F | USA | Control | 9,7 | 3,66 |
| F | USA | CuSO_4_ | 0,0 | 0,0 |
| F | USA | Elevated salinity | 0,1 | 0,05 |
| F | USA | Elevated salinity x CuSO_4_ | 0,0 | 0,0 |

Figure S1: Unrooted SNP-based phylogeny of the six *Daphnia magna* genotypes investigated in this study*.* Bootstraps are indicated at each branch point. Scale indicates 0.1 substitutions per site. Genotype sites were filtered to select for biallelic SNPs of GQ ≥ 30, and to remove non-variant sites and unused alternates using GATK v. 4.6.2.0 (van der Auwera and O’Connor, 2020). Sites with minor allele frequencies < 0.05, or missingness > 0.05 were removed using BCFTools v. 1.22 (Danecek et al., 2021) and Plink2 v.2.0.0-a (Chang et al., 2015). Site imputation was performed using Beagle v.5.5 (Browning et al., 2018). Pairwise independent sites (r­^2^ < 0.2) were then selected for using Plink2. The sites for the six genotypes of interest were subset, and converted to PHYLIP format using vcf2phylip v.2.8 (Ortiz, 2019). The phylogeny was generated using RAxML-NG v.1.2.2 (Kozlov et al., 2019) using the GTR gamma model of substitution and 100 bootstraps. This tree was visualised using the Interactive Tree of Life (iTOL) (Letunic and Bork, 2021).


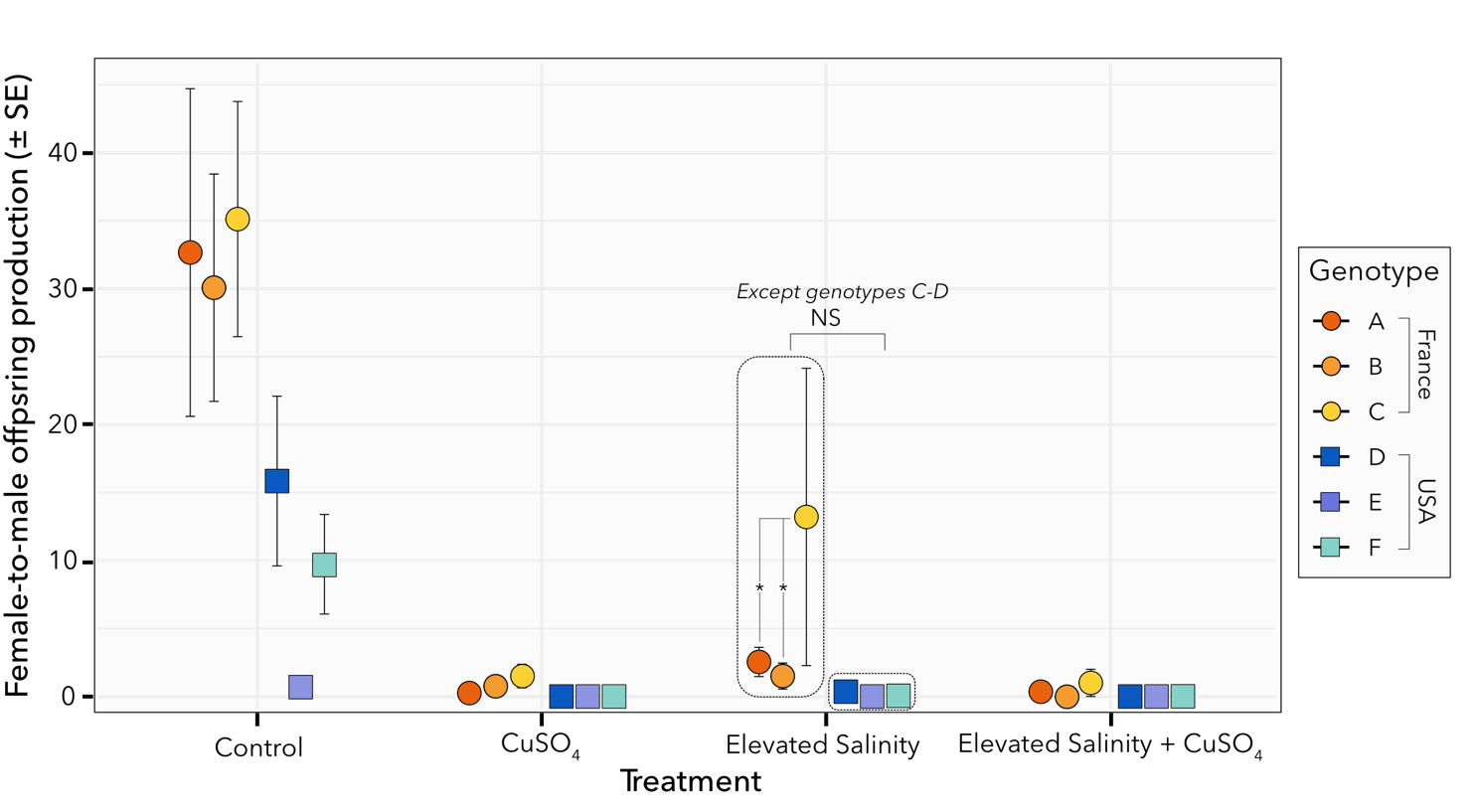


Figure S2: Female-to-male offspring production (± SE) of *Daphnia magna* across experimental conditions: Control, CuSO_4_, Elevated salinity and CuSO_4_ + Elevated Salinity. Results are shown for the six genotypes (A to F) from two locations – France (genotypes A, B, C) and USA (genotypes D, E, F). In elevated salinity conditions, genotypes from France did not differ from genotypes from the USA, except for genotypes C and D, who responded differently.

**References**

Browning, B.L., Zhou, Y., Browning, S.R., 2018. A one-penny imputed genome from next-generation reference panels. Am. J. Hum. Genet. 103, 338–348. <https://doi.org/10.1016/j.ajhg.2018.07.015>

Chang, C.C., Chow, C.C., Tellier, L.C., Vattikuti, S., Purcell, S.M., Lee, J.J., 2015. Second-generation PLINK: rising to the challenge of larger and richer datasets. Gigascience 4. <https://doi.org/10.1186/s13742-015-0047-8>

Kozlov, A.M., Darriba, D., Flouri, T., Morel, B., Stamatakis, A., 2019. RAxML-NG: a fast, scalable and user-friendly tool for maximum likelihood phylogenetic inference. Bioinformatics 35, 4453–4455. <https://doi.org/10.1093/bioinformatics/btz305>

Letunic, I., Bork, P., 2021. Interactive tree of life (iTOL) v5: an online tool for phylogenetic tree display and annotation. Nucleic Acids Res. 49, W293–W296. <https://doi.org/10.1093/nar/gkab301>

Ortiz, E.M., 2019. vcf2phylip v2.0: convert a VCF matrix into several matrix formats for phylogenetic analysis. <https://doi.org/10.5281/ZENODO.2540861>

van der Auwera, G.A., O’Connor, B.D., 2020. Genomics in the cloud: using Docker, GATK, and WDL in terra, First edition. ed. O’Reilly, California.
